# Synthesis and Structure of Diphosphene-Bridged Dicarborane Dianions with [2*n*+3] Skeletal Electrons

**DOI:** 10.1021/acs.inorgchem.5c04262

**Published:** 2025-12-22

**Authors:** Tek Long Chan, Jie Zhang, Zuowei Xie

**Affiliations:** † Department of Chemistry and State Key Laboratory of Synthetic Chemistry, 26451 The Chinese University of Hong Kong, Shatin, New Territories, Hong Kong, China; ‡ Shenzhen Grubbs Institute and Department of Chemistry, Southern University of Science and Technology, Shenzhen, Guangdong 518055, China

## Abstract

In contrast to the extensively studied carboranes with even skeletal electron counts ([2*n*+2], [2*n*+4]), systems with odd electron counts ([2*n*+3]) are rare (*n* = number of skeletal atoms). With the assistance of a diphosphene-bridge, dicarborane dianions {[(Ar)­NC­(^t^Bu)­C_2_B_10_H_10_]­P}_2_^2–^ [Ar = Dipp (**2**) or Dmp (**4**); Dipp = 2,6-^i^Pr_2_C_6_H_3_, Dmp = 2,6-Me_2_C_6_H_3_] with formal [2*n*+3] skeletal electrons in each carborane cage were obtained via single electron reduction of imine-stabilized carboranyl-phosphinidene or -diphosphene with potassium graphite (KC_8_) or cobaltocene (CoCp_2_), respectively. The delocalization of the two unpaired electrons across the carborane cages and diphosphene-bridge was evidenced by experimental and theoretical results.

## Graphical abstract

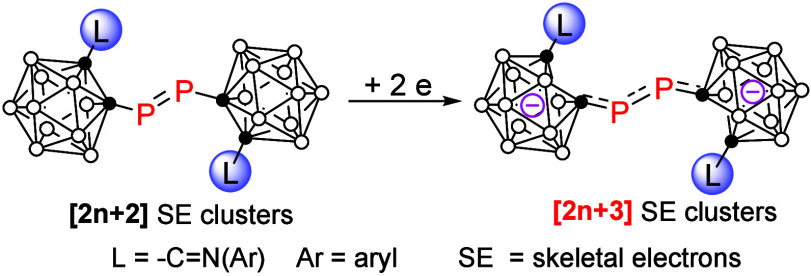

Icosahedral carboranes, a class of carbon-containing borane clusters, are well-known for their exceptional thermal stability, a nearly spherical architecture, and extensive three-dimensional delocalization of σ-bonding electrons.^1^ According to the electron-counting rules (Wade’s rules), *closo*-carborane clusters are stabilized by a total of [2*n*+2] skeletal electrons.^2^ When *closo*-carboranes undergo two-electron reduction by alkali metals, the resulting *nido*-carboranes adopt a more open framework consistent with a [2*n*+4] skeletal electron configuration.^3^ In contrast, clusters possessing [2*n*+3] skeletal electrons, which occupy an intermediate electron count between the *closo* [2*n*+2] and *nido* [2*n*+4] structural systems, are relatively rare. So far, only two structurally characterized examples of carborane anions with [2*n*+3] skeletal electrons are known. One is a 13-vertex carborane radical monoanion [·{1,2-(CH_2_)_3_-1,2-C_2_B_11_H_11_}]­[Na­(18-crown-6)­(THF)_2_] established by the Xie group in 2007 (Figure 1A).^4^ The other one was reported by the Fox group in 2014, where the 12-vertex dicarborane dianions with a formal [2*n*+3] skeletal electron count in each carborane cluster were stabilized through *para*-phenylene π-conjugation (Figure 1B).^5^

**Figure 1:**
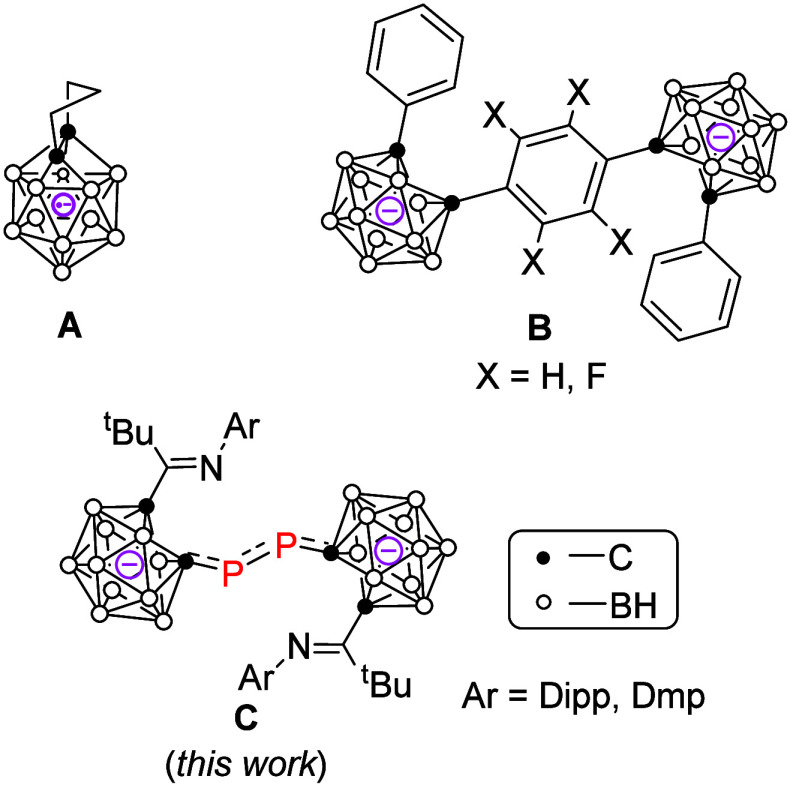
Carborane anions with (formal) [2*n*+3] skeletal electrons. Dipp = 2,6-^i^Pr_2_C_6_H_3_, Dmp = 2,6-Me_2_C_6_H_3_.

Driven by their utility in organic synthesis, biological process, and materials science, the synthesis of highly reactive phosphorus-centered radicals is an area of growing investigation.^6^ A number of phosphorus-based radical species containing two phosphorus atoms, stabilized by sterically demanding ligands and spin delocalization across π-conjugated frameworks, have been isolated and characterized.^7,8^ In 2017, the Yamashita group disclosed radical anions featuring two boryl substituents that act as strong σ-donors and π-acceptors, enabling extensive delocalization of the unpaired electron across the B–P=P–B framework.^8^

Recently, we have reported the synthesis, structure, and reactivity of imine-stabilized carboranyl phosphinidene and diphosphenes.^9^ Diphosphenes are known to accommodate one additional electron at the P=P bond, resulting in the formation of a radical anion species.^8,10^ We wondered if the single-electron reduction of the phosphinidene or diphosphene could afford the phosphorus-center radical anions, followed by delocalization of the spin electron to access the carborane cluster with formal [2*n*+3] skeletal electrons. With this in mind, single-electron reduction of imine-stabilized carboranyl phosphinidene and diphosphene was conducted, resulting in the isolation of diphosphene-bridged dicarborane dianions with formal [2*n*+3] skeletal electrons in each cage (Figure 1C). These results are reported in this Communication.

Treatment of iminocarboranyl phosphinidene **1** with 1 equiv of potassium graphite (KC_8_) and 18-crown-6 ether in THF immediately gave a deep blue solution from which dicarborane dianionic salt [**2**]­[K]_2_ was obtained as deep blue crystals in 75% yield via recrystallization from a mixed solvent of toluene and THF (Scheme 1a). On the other hand, reduction of carboranyl diphosphene **3** with 2 equiv of cobaltocene (CoCp_2_) in ether resulted in a deep green solution from which [**4**]­[CoCp_2_]_2_ was isolated as greenish blue crystals in 70% yield (Scheme 1b). The phosphorus chemical shifts of [**2**]^2–^ and [**4**]^2–^ were observed at 345.8 and 330.3 ppm, respectively, which was downfield shifted in comparison with that of ca. 210 ppm in compounds **1** and **3**. This observation might be ascribed to the delocalization of these systems.

**Scheme 1:**
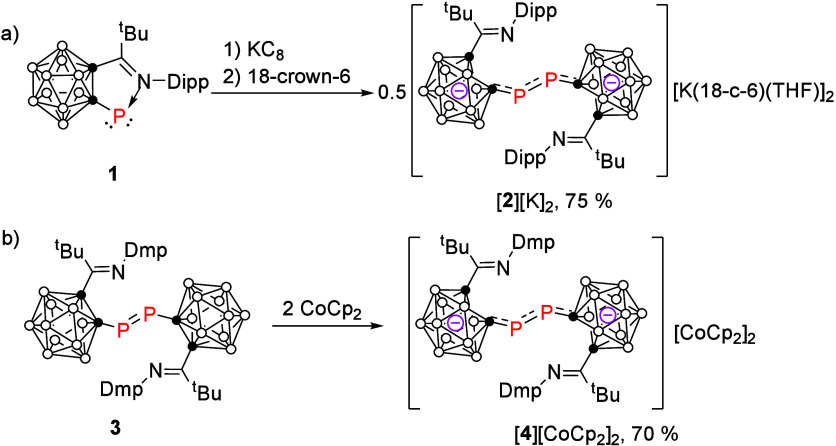
Synthesis of the Dicarborne Dianionic Salts [**2**]­[K]_2_ and [**4**]­[CoCp_2_]_2_

Single-crystal X-ray diffraction analyses unambiguously confirmed the molecular structures of [**2**]^2–^ and [**4**]^2–^, as illustrated in Figures 2 and 3, respectively. The P atoms in [**2**]^2–^ and [**4**]^2–^ form σ bonds to one C_cage_ and one P atom with C2–P1–P1# angles of 100.7(1)° and 100.9(1), respectively. The P1–P1# bond distances of 2.139(1) Å in [**2**]^2–^ and 2.143(1) Å in [**4**]^2–^ fall in between those observed in reported carbon-substituted diphosphenes [1.985(3)–2.051(2) Å] ^9b,11^ and diphosphane compounds [2.234(4)–2.300(1) Å].^12^ The significantly shorter P1–C2 distances in [**2**]^2–^ (1.733(2) Å) and [**4**]^2–^ (1.731(2) Å), compared to those in **1** (1.811(4) Å) and **3** (1.871(2) Å), indicate exo π-bonding character in the cage C–P bonds. The C1–C2 distances in [**2**]^2–^ (2.416 Å) and in [**4**]^2–^ (2.396 Å) were comparable to those (2.370–2.416 Å) in dicarborane dianion [1,4-(PhC_2_B_10_H_10_)_2_C_6_H_4_]^2–^,^5^ biscarborane dianion,^13^ and [1-(C_6_H_5_CH_2_)-2-(C_6_H_5_CH)-1,2-C_2_B_10_H_10_]­[(THF)­K­(18-crown-6)][Bibr ref14] but significantly longer than that of 1.637(4) Å in **1** and 1.730(2) Å in **3**, respectively, which were consistent with that of the opened carborane cage. These data suggested that the additional two electrons have been delocalized over the carborane cages and diphosphene-bridge via *exo* π-bonding interaction.

**Figure 2:**
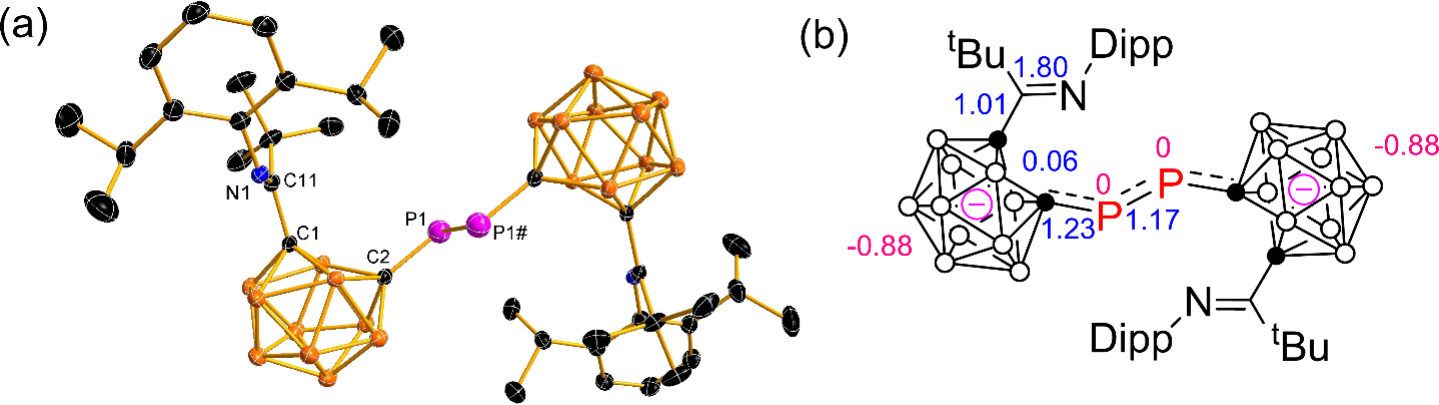
(a) Solid-state structure of the anion [**2**]^2–^. Selected bond distances (Å) and angles (°): P1–P1# 2.139(1), C1–C2 2.416, P1–C2 1.733(2), C11–N1 1.269(2), C11–C1 1.523(2); C2–P1–P1# 100.7(1), N1–C11–C1 112.7(2), C11–C1–C2 126.0, C1–C2–P1 121.6. (b) Natural population analysis (NPA) charges (in red) for selected atoms and Wiberg bond indices (WBI) (in blue) for selected bonds in [**2**]^2–^.

**Figure 3:**
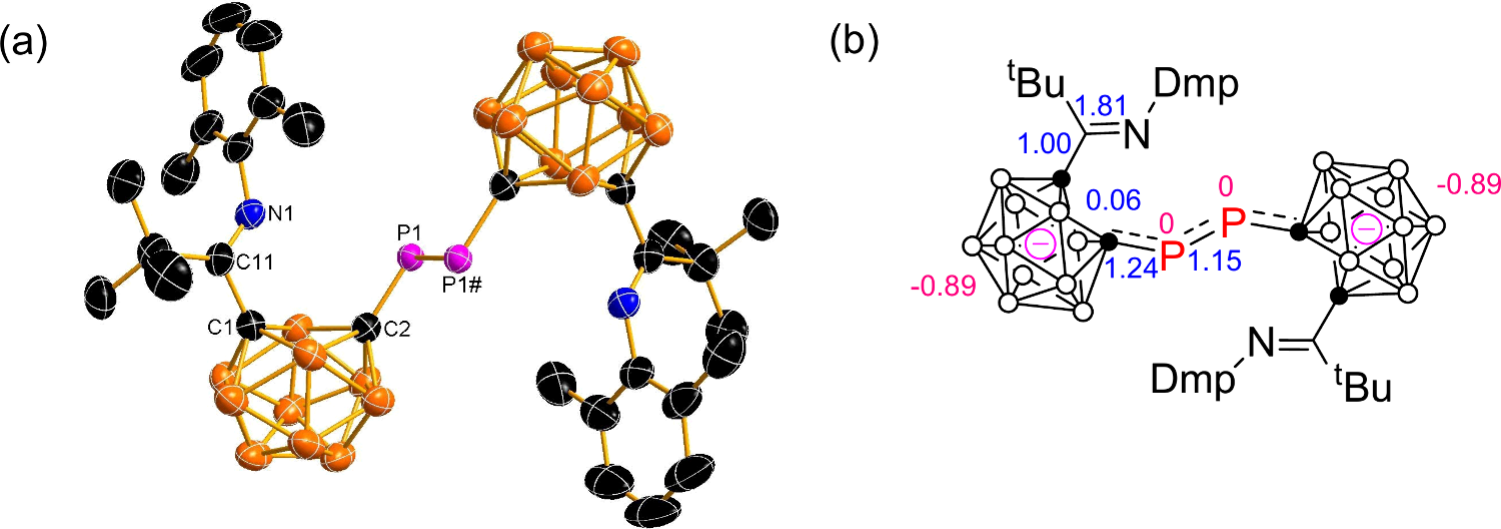
(a) Solid-state structure of [**4**]^2–^. Selected bond distances (Å) and angles (°): P1–P1# 2.143(1), C1–C2 2.396, P1–C2 1.731(2), C11–N1 1.262(2), C11–C1 1.512(3); C2–P1–P1# 100.9(1), N1–C11–C1 112.4(2), C11–C1–C2 117.0, C1–C2–P1 120.6. (b) Natural population analysis (NPA) charges (in red) for selected atoms and Wiberg bond indices (WBI) (in blue) for selected bonds in [**4**]^2–^.

Then, density functional theory (DFT) calculations on [**2**]^2–^ and [**4**]^2–^ were performed, and the selected frontier molecular orbitals (FMOs) are illustrated in Figure 4. The LUMOs (lowest unoccupied molecular orbital) of the anion [**2**]^2–^ and [**4**]^2–^ represented the antibonding orbitals delocalized over the two carborane cages and the P=P bridge. The HOMOs (highest occupied molecular orbitals) of the anions [**2**]^2–^ and [**4**]^2–^ consist of π orbitals of the P=C_cage_ bond, revealing that the electron density is delocalized over the C_cage–P=P–C_cage unit. The HOMO-2 of [**2**]^2–^ and HOMO-4 of [**4**]^2–^ mainly consist of the π (P=P) orbitals with some contributions from the aryl ring. The results agree with the observed elongation of the P=P bond, together with a shortening of the C–P bonds in [**2**]^2–^ and [**4**]^2–^. On the other hand, the Wiberg bond indices (WBIs) and charges on the two separated carborane cages of anions [**2**]^2–^ and [**4**]^2–^ are similar to each other as indicated in Figures 2 and 3, respectively (for details, see Table S2 in the SI). The bond orders of 1.23–1.24 for the cage C–P bond and 1.15–1.17 for the P–P bond are consistent with the partially multiple-bonding nature of these two bonds. The diphosphene bridges in [**2**]^2–^ and [**4**]^2–^ exhibit nearly zero NPA charges, indicating a neutral character. These results resemble those of [1,4-(PhC_2_B_10_H_10_)_2_C_6_H_4_]^2–^,^5^ confirming a formal [2*n*+3] skeletal electron count for each cage in the dianions.

**Figure 4:**
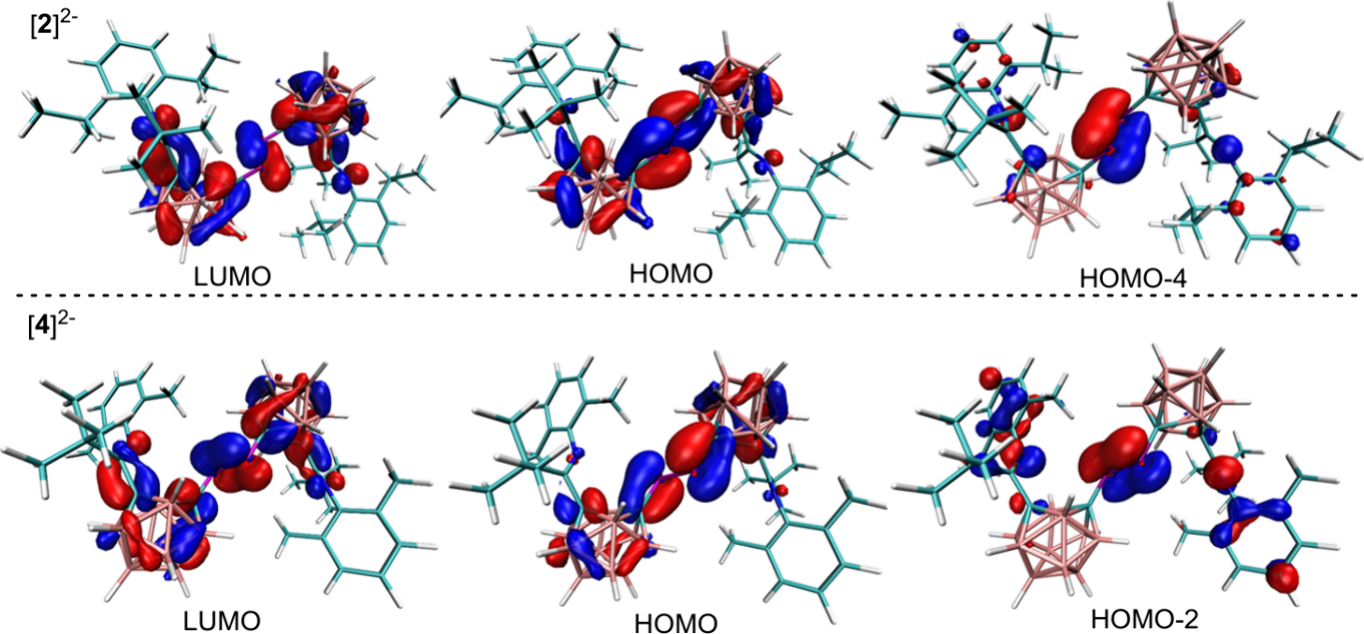
Selected FMOs of [**2**]^2–^ (top) and [**4**]^2–^ (bottom) calculated at B3LYP/6-311++G­(d,p) level of theory.

To probe the reduction pathway, we wondered if the iminocarboranyl ligand plays a role for the generation of anionic cluster with [2*n*+3] skeletal electrons. Reduction of **5**, which was prepared by reaction of iminocarboranyl lithium with phenylphosphine dichloride (PhPCl_2_), with 1 equiv of potassium graphite (KC_8_) in THF gave dicarboranyl-diphosphane **6** in 80% yield (Scheme 2). Compound **6** displays a ^31^P NMR resonance at 26.5 ppm, representing a significant upfield shift relative to its precursor **5**, which exhibits a resonance at 73.0 ppm. Compound **6** can be viewed as the dimer of the carboranyl-phosphorus­(II) radical. Unfortunately, treatment of **6** with 2 or 4 equiv agents, such as KC_8_, sodium naphthalide, and lithium naphthalide, in THF could not give further reduction products. These results demonstrated that the phosphinidene or diphosphene moiety, instead of the imino group on the cage C vertex, played a crucial role in the single-electron reduction of the carborane cages, leading to the opened clusters with formal [2*n*+3] skeletal electrons per cage. Moreover, cyclic voltammetry of a THF solution of **1** and **3** (1 mM, supporting electrolyte 0.1 M Bu_4_NPF_6_, scan rate 50 mV/s) showed a quasi-reversible peak at *E*_1/2_ = −1.32 V and −1.58 V (versus Fc/Fc^+^), respectively, comparable to that observed in 1,4-bis­(2′-phenyl-*o*-carboran-1′-yl)­benzenes (*E*_1/2_ = −1.56 V).^5^ In contrast, only one irreversible peak at *E* = −1.81 V (versus Fc/Fc^+^) was observed for **6** (Figure 5).

**Figure 5:**
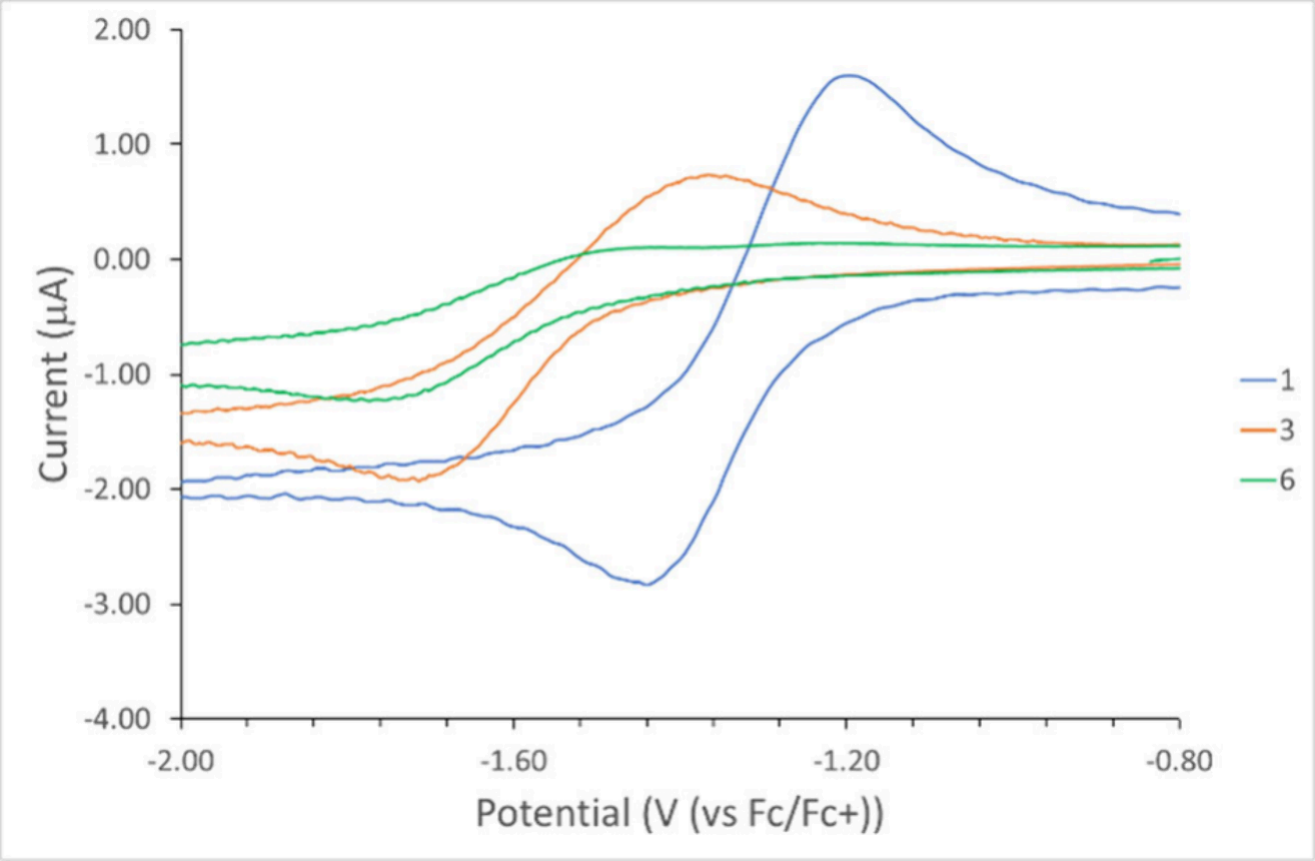
Cyclic voltammogram of **1**, **3**, and **6** (1 mM in 0.1 M Bu_4_NPF_6_/THF, scan rate 50 mV/s, potential versus Fc/Fc^+^, scanned from −2.0 to 1.0 V). Electrochemical measurements were conducted using a 3 mm glassy carbon disk as the working electrode, a platinum wire as the auxiliary electrode, and a silver wire as the pseudoreference electrode.

**Scheme 2:**
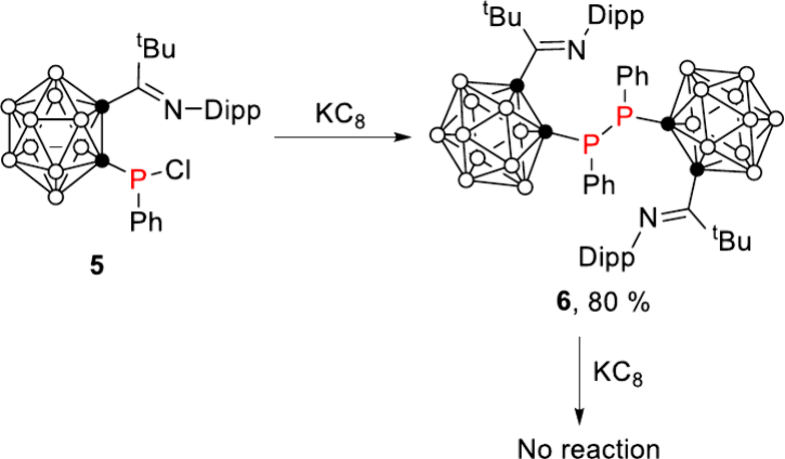
Preparation of Dicarboranyl Diphosphane **6**

Single-crystal X-ray diffraction analysis revealed the molecular structure of compound **6**, as illustrated in Figure 6. The P1 atom in **6** exhibits a trigonal pyramidal geometry, forming three σ bonds with one cage C atom, one phenyl C atom, and one P atom, respectively. The P1–C2 distance of 1.907(5) Å is significantly longer than that in **2** [1.733(2) Å]. The P1–P2 bond length of 2.304(2) Å lies within the characteristic range reported for diphosphanes [2.234(4)–2.300(1) Å].^12^

**Figure 6:**
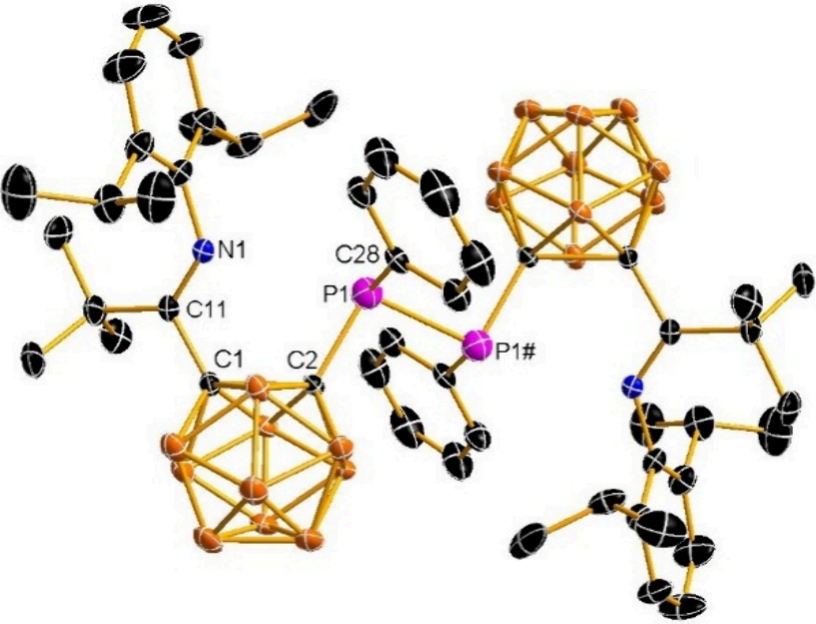
Solid-state structure of **6**. Selected bond distances (Å) and angles (°): C1–C2 1.825(6), P1–C2 1.907(5), P1–C28 1.853(5), P1–P1# 2.304(2), C11–N1 1.270(6), C11–C1 1.550(7); C2–P1–P1# 98.9(2), N1–C11–C1 111.9(4), C11–C1–C2 119.5(4), C1–C2–P1 118.3(3).

Based on these results, a mechanism for the preparation of [**2**]^2–^ was proposed (Scheme 3). The monomeric carboranyl phosphinidene accepts an electron from KC_8_ to generate the radical anion intermediate **A**. Formation of the *exo* π-bonding between phosphorus and cage carbon atom leads to the cleavage of cage carbon–carbon bond^15^ to give **B** featuring [2*n*+3] skeletal electrons. Dimerization of **B** affords the dianionic product [**2**]^2–^.

**Scheme 3:**
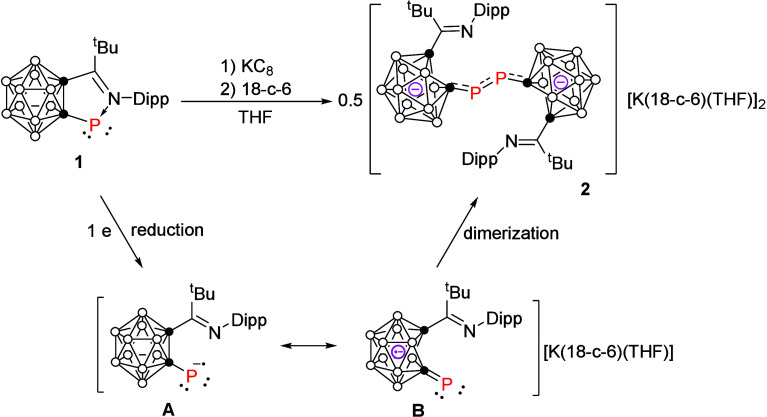
Proposed Mechanism for the Generation of [**2**]^2–^

In summary, dicarborane dianions [**2**]^2–^ and [**4**]^2–^ featuring a diphosphene-bridge with [2*n*+3] skeletal electrons in each carborane cluster have been prepared by single-electron reduction of imine-stabilized carboranyl phosphinidene **1** and diphosphene **3**, respectively. Upon treatment of **1** or **3** with reductant, the single electron on the phosphorus center was delocalized into the electron-deficient carborane cage via an exo π-interaction between P and C_cage_ atom, simultaneously cleaving the C_cage_–C_cage_ bond to result in a carborane anion with [2*n*+3] skeletal electrons. Such π conjugation over the diphosphene-bridging unit offers stabilization to this rare [2*n*+3] system.

## Supplementary Material



## Notes

Experimental details, characterizations, computational details, and NMR spectra (PDF)

## Notes

*Caution!* No uncommon hazards are noted.
