# Supplementary material for: Synthesis and Structure of Diphosphene-Bridged Dicarborane Dianions with [2n+3] Skeletal Electrons
Source: Inorg Chem. 2025 Dec 22;65(1):7–12. doi: 10.1021/acs.inorgchem.5c04262 (PMC12801381; doi:10.1021/acs.inorgchem.5c04262)
Supplement: Supplementary file 1 [file ic5c04262_si_001.pdf]

Supporting Information

Synthesis and Structure of Diphosphene-Bridged Dicarborane Dianions with [2n+3] Skeletal Electrons

Tek Long Chan,[a] Jie Zhang[a] and Zuowei Xie*[b]*

[a] Department of Chemistry, The Chinese University of Hong Kong, Shatin, New Territories, Hong Kong, China.

[b] Shenzhen Grubbs Institute and Department of Chemistry, Southern University of Science and Technology, Shenzhen, Guangdong 518055, China

Email: jiezhang@cuhk.edu.hk; zxie@cuhk.edu.hk

Table of Contents

Experimental Section S2

Crystal Data and Summary of Data Collection and Refinement S6

Computational Details S8

References S18

NMR Spectra S19

General Procedures. All operations were carried out under a dry argon atmosphere using standard Schlenk and glovebox techniques. ^{1}H, ^{13}C, ^{11}B and ^{31}P NMR spectra were recorded on a Bruker DPX 400 spectrometer or Bruker DPX 500 spectrometer at 400 or 500, 100 or 125, 128 or 160 and 162 or 202 MHz, respectively. All chemical shifts were reported in δ units with references to the residual solvent resonances of the deuterated solvents for proton and carbon chemical shifts, to external $BF_3{\cdot}OEt_2$ (0.00 ppm) for boron chemical shifts, and to 85% phosphoric acid (H_3PO_4) for phosphorus chemical shifts. NMR multiplicities were abbreviated as follows: s = singlet, d = doublet, t = triplet, m = multiplet, br = broad signal. Mass spectra were obtained on a Thermo Finnigan MAT 95 XL spectrometer. Cyclic voltammetry was performed on a PAR Potentiostat / Galvanostat Model 263A Electrochemical Station (Princeton Applied Research) at a scan rate of 50 mV/s. A 3 mm glassy carbon disc was used as a working electrode, a platinum wire was used as an auxiliary electrode, and a silver wire was used as a pseudo-reference. UV-Visible absorption spectra were recorded on a Varian Cary5G UV-Vis-NIR spectrophotometer (in the range of 200-1100 nm) or Hitachi UH5300 spectrometer (in the range of 190-1100 nm) using 1 cm quartz cells under argon atmosphere. Elemental analyses were performed by MEDAC Ltd, U.K., or the Shanghai Institute of Organic Chemistry, CAS, China. All organic solvents were freshly distilled from sodium benzophenone ketyl immediately prior to use. **1**,[1] **3**[2] and 1-C(^{t}Bu)=N(Dipp)-1,2-$C_2B_{10}H_{11}$[3] were prepared according to literature procedures. All other chemicals were purchased from Aldrich, J&K or Acros Chemical Co. and used as received unless otherwise specified.

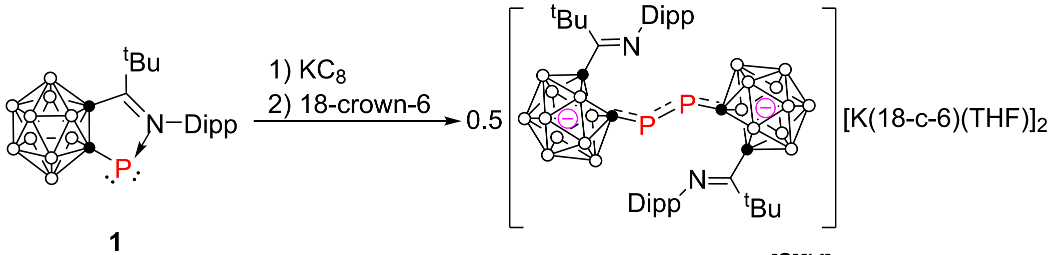

Preparation of [2][K]$_2$. A THF solution (20 mL) of **1** (83.4 mg, 0.2 mmol) and 18-crown-6 ether (52.8 mg, 0.2 mmol) was slowly added to a mixture of KC_8 (27.0 mg, 0.2 mmol) in THF at room temperature. After stirring overnight at room temperature, the color of the solution was changed to navy blue. After filtration, the blue filtrate was concentrated to about 3 mL. Compound [**2**][K]$_2$ was isolated as navy blue crystals after the solution stood at room temperature overnight (119.0 mg, 75 %). ^{1}H NMR (400 MHz, CD_3CN): δ 6.94 (d, J = 7.4 Hz, 4H), 6.75 (t, J = 7.4 Hz, 2H) (aromatic C*H*), 3.66 (m, 8H) (THF), 3.59 (s, 48H) (18-c-6), 2.82 – 2.78 (m, 4H) (C*H*Me$_2$), 1.82 (m, 8H) (THF), 1.14 (d, J =

6.5 Hz, 12H) (CH*Me*$_2$), 1.11 (s, 18H) (C*Me*$_3$), 1.08 (d, *J* = 6.5 Hz, 12H) (CH*Me*$_2$). $^{13}C\{^1H\}$ NMR (100 MHz, CD_3CN): δ 170.5 (*C*=N), 148.3, 132.8, 122.8, 120.9 (aromatic *C*), 70.9, 68.3, 45.9, 30.1, 28.6, 26.2, 25.1, 22.5, cage *C* was not observed. $^{11}B\{^1H\}$ NMR (128 MHz, CD_3CN): δ -0.5 (2B), -6.1 (12B), -10.9 (2B), -23.4 (4B). ^{31}P NMR (162 MHz, CD_3CN): δ 345.8. Anal. Calcd for $C_{66}H_{128}B_{20}K_2N_2O_{13}P_2$ ([**2**][K]$_2$ −THF): C 52.36, H 8.52, N 1.85. Found: C 52.23, H 8.44, N 1.85.

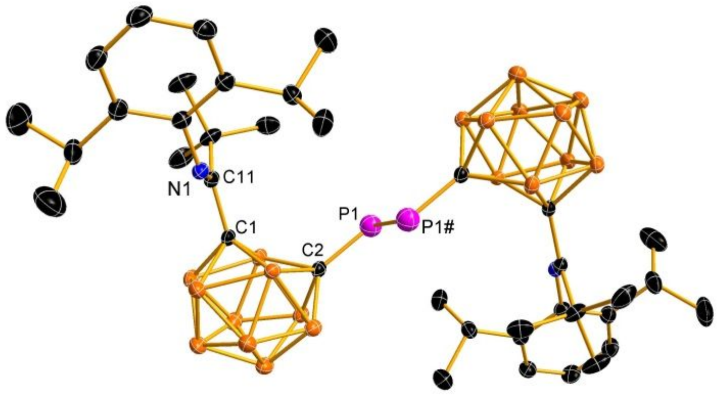

Figure S1. Molecular structure of [**2**]$^{2-}$.

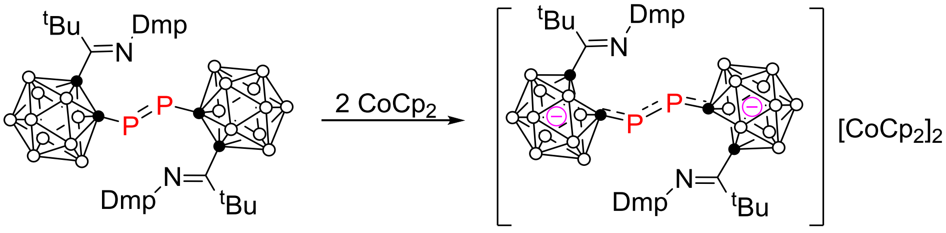

3

[**4**][$CoCp_2$]$_2$

Preparation of [4][CoCp$_2$]$_2$. An ether solution (20 mL) of **3** (72.2 mg, 0.1 mmol) was slowly added to Cp_2Co (37.8 mg, 0.2 mmol) in diethyl ether (10 mL) at room temperature, and the reaction mixture was stirred overnight at room temperature. The color of the solution was changed to dark brown. After filtration, the dark green solution was concentrated to about 3 mL. Compound [**4**][$CoCp_2$]$_2$ was isolated as greenish blue crystals after the solution stood at room temperature overnight (77.1 mg, 70 %). ^1H NMR (400 MHz, CD_3CN): δ 6.83 (d, *J* = 7.4 Hz, 4H), 6.59 (t, *J* = 7.4 Hz, 2H) (aromatic C*H*), 1.98 (s, 12H) (Ar*Me*), 1.08 (s, 18H) (C*Me*$_3$), -1.93 (br s, 20H) (*Cp*$_2$Co). $^{13}C\{^1H\}$ NMR (125 MHz, CD_3CN): δ 169.1 (*C*=N), 151.4, 128.4, 127.7, 122.7, 120.0 (aromatic *C*), 46.3, 29.2, 19.3, cage *C* and *Cp*$_2$Co$^-$ *C* were not observed. $^{11}B\{^1H\}$ NMR (128 MHz, CD_3CN): δ -1.5 (4B), -8.1

(12B), -10.9 (2B), -24.3 (2B). ^{31}P NMR (202 MHz, CD_3CN): δ 330.3. Anal. Calcd for $C_{50}H_{76}B_{20}Co_2N_2P_2$: C 54.54, H 6.96, N 2.54. Found: C 54.31, H 6.87, N 3.07.

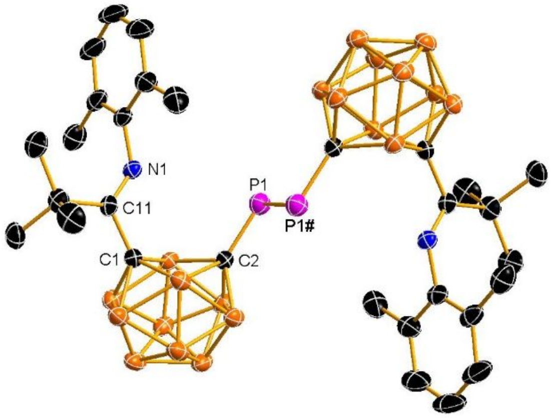

Figure S2. Molecular structure of [**4**]$^{2-}$.

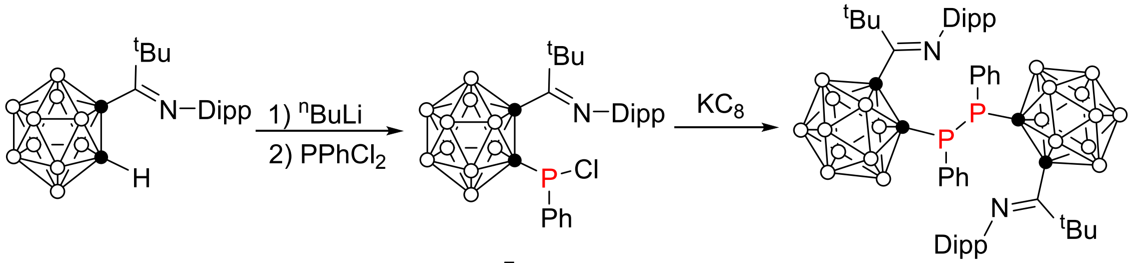

5 **6**

Preparation of 5. To an ether solution (20 mL) of 1-C(^{t}Bu)=N(Dipp)-1,2-$C_2B_{10}H_{11}$ (0.930 g, 2.4 mmol) was slowly added via syringe a hexane solution of n-BuLi (1.6 M, 1.50 mL, 2.4 mmol) at 0 ^{o}C under stirring. The reaction mixture was allowed to warm up to room temperature and stirred overnight. $PPhCl_2$ (430 mg, 2.4 mmol) was then added dropwise at room temperature. The resulting mixture was stirred for 4 h. After removal of the precipitate by filtration, the filtrate was concentrated to about 5 mL, to which was added n-hexane (2 mL). Compound **5** was isolated as yellow crystals after the solution stood at room temperature overnight (1.020 g, 80 %). ^{1}H NMR (400 MHz, CD_2Cl_2): δ 7.52 (t, J = 8.0 Hz, 2H), 7.42 (t, J = 7.2 Hz, 1H), 7.30 (t, J = 7.6 Hz, 2H), 7.17 (d, J = 7.6 Hz, 1H), 7.11 (t, J = 7.6 Hz, 1H), 7.02 (d, J = 7.6 Hz, 1H) (aromatic C*H*), 2.77-2.84 (m, 1H), 2.43-2.50 (m, 1H) (C*H*Me$_2$), 1.35 (d, J = 6.8 Hz, 6H) (CH*Me*$_2$), 1.30 (s, 12H) (C*Me*$_3$ and CH*Me*$_2$), 1.19 (d, J = 6.8 Hz, 3H) (CH*Me*$_2$), 0.75 (d, J = 6.8 Hz, 3H) (CH*Me*$_2$). ^{13}C{^{1}H} NMR (100 MHz, CD_2Cl_2): δ 164.2 (*C*=N), 142.6, 139.2, 138.8, 135.2, 132.3, 132.0, 131.8, 128.9, 128.8, 124.8, 124.2, 124.1 (aromatic *C*), 87.6 (d, $^2J_{PC}$ = 49.2 Hz, cage *C*), 87.1 (d, $^1J_{PC}$ = 55.6 Hz, cage *C*), 46.6 (*C*Me$_3$), 30.5 (*C*HMe$_2$), 28.9, 28.6, 26.4, 25.9, 23.7 (C*Me*$_3$, CH*Me*$_2$). ^{11}B{^{1}H} NMR (128 MHz, CD_2Cl_2): δ -1.8 (2B), -8.4 (4B), -10.6 (4B). ^{31}P NMR (162

MHz, CD_2Cl_2): δ 73.0. Anal. Calcd for $C_{25}H_{41}B_{10}ClNP$: C 56.64, H 7.80, N 2.64. Found: C 56.15, H 7.65, N 2.96.

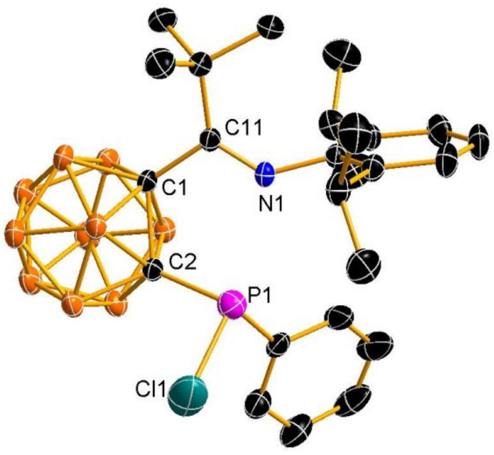

Figure S3. Molecular structure of **5**.

Preparation of 6. A THF solution (20 mL) of **5** (635 mg, 1.2 mmol) was slowly added to a mixture of KC_8 (162 mg, 1.2 mmol) in THF at room temperature, and the reaction mixture was stirred overnight at room temperature. The color of the solution was changed to orange yellow. After filtration, the orange yellow filtrate was concentrated to about 5 mL. Compound **6** was isolated as orange yellow crystals after the solution stood at room temperature overnight (474 mg, 80 %). ^{1}H NMR (500 MHz, $CDCl_3$): δ 7.20 (d, *J* = 7.4 Hz, 2H), 7.06 (t, *J* = 7.5 Hz, 2H), 7.00 (d, *J* = 7.1 Hz, 6H), 6.81 (t, *J* = 7.2 Hz, 2H), 6.52 (d, *J* = 6.6 Hz, 4H) (aromatic C*H*), 3.55 – 3.53 (m, 2H), 2.29 – 2.26 (m, 2H) (C*H*Me_2), 1.53 (d, *J* = 6.2 Hz, 6H), 1.40 (d, *J* = 6.4 Hz, 6H) (CHMe_2), 1.31 (s, 18H) (CMe_3), 1.12 (d, *J* = 6.6 Hz, 6H), 0.63 (d, *J* = 6.0 Hz, 6H) (CHMe_2).$^{13}C\{^{1}H\}$ NMR (125 MHz, $CDCl_3$): δ 162.5 (*C*=N), 144.5, 137.1, 134.5, 133.4, 129.6, 126.9, 124.1, 123.5, 122.9 (aromatic *C*), 91.8 (cage *C*), 47.3, 30.7, 28.7, 27.9, 27.5, 24.6, 23.9, 22.8, another cage *C* was not observed. $^{11}B\{^{1}H\}$ NMR (160 MHz, $CDCl_3$): δ -0.5 (4B), -5.3 (4B), -7.3 (4B), -9.6 (8B). ^{31}P NMR (202 MHz, $CDCl_3$): δ 26.5. HRMS: m/z calcd for $(C_{50}H_{82}{}^{10}B_4{}^{11}B_{16}N_2P_2+Na)^+$: 1011.7852. Found: 1011.7853.

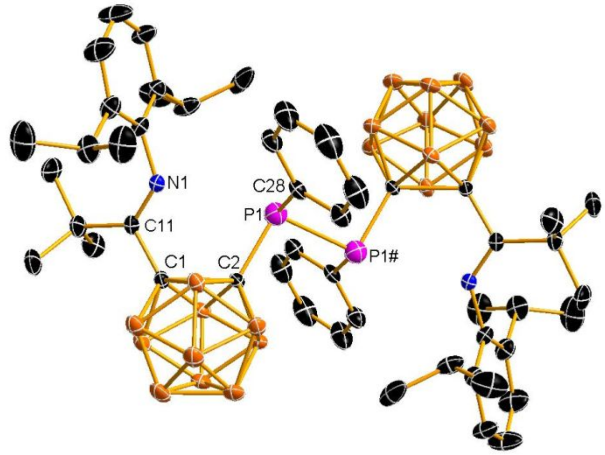

Figure S4. Molecular structure of **6**.

X-ray Structure Determination. Single crystals were immersed in Paraton-N oil and sealed under argon in thin-walled glass capillaries. All data were collected at 296 K or 173 K on a Bruker D8 venture diffractometer or a Bruker Kappa ApexII Duo diffractometer using Mo-Kα radiation. An empirical absorption correction was applied using the SADABS program.[4] All structures were solved by direct methods and subsequent Fourier difference techniques and refined anisotropically for all non-hydrogen atoms by full-matrix least squares calculations on F^2 using the SHELXTL program package.[5] All hydrogen atoms were geometrically fixed using the riding model. Crystal data and details of data collection and refinement are given in Table S1. Details of the crystal structures were deposited in the Cambridge Crystallographic Data Centre with CCDC 2479758–2479761 for [**2**][K]$_2$, [**4**][$CoCp_2$]$_2$, **5** and **6**.

Table S1. Crystal Data and Summary of Data Collection and Refinement for [**2**][K]$_2$, [**4**][CoCp$_2$]$_2$, **5** and **6**

Compound	[**2**][K]$_2$	[**4**][CoCp$_2$]$_2$	**5**	**6**
Formula	$C_{70}H_{136}B_{20}K_2N_2O_{14}P_2$	$C_{50}H_{76}B_{20}Co_2N_2P_2$	$C_{25}H_{41}B_{10}ClNP$	$C_{50}H_{82}B_{20}N_2P_2$
MW	1586.14	1101.12	530.11	989.31
Crystal size (mm^3)	0.50×0.30×0.20	0.40×0.30×0.20	0.50×0.40×0.30	0.50×0.40×0.30
Crystal system	Triclinic	Triclinic	Triclinic	Monoclinic
Space Group	*P*-1	*P*-1	*P*-1	$P2_1/c$
a, Å	12.115(1)	10.634(1)	11.279(1)	15.633(1)
b, Å	12.237(1)	12.176(1)	11.818(1)	16.205(1)
c, Å	17.078(1)	13.533(1)	12.462(1)	12.318(1)
α, deg	106.08(1)	81.5904(15)	75.90(1)	90
β, deg	93.84(1)	83.6981(16)	71.47(1)	106.10(1)
γ, deg	94.64(1)	89.5474(15)	82.66(1)	90
V, Å^3	2414.3(1)	1722.9(1)	1525.2(2)	2998.0(3)
Z	1	1	2	2
D_{calcd} mg/m^3	1.091	1.061	1.154	1.096
Radiation (Å)	0.71073	0.71073	1.54178	0.71073
2θ range, deg	2.49 to 56.09	3.38 to 55.82	7.66 to 137.47	2.71 to 50.50
μ, mm^{-1}	0.184	0.559	1.702	0.108
F(000)	850	574	560	1052
No. of obsd reflns	11334	8199	5599	5426
No. of params refnd	496	343	344	334
Goodness of fit	1.032	1.050	1.057	1.166
R1	0.0524	0.0487	0.0414	0.0983
wR2	0.1196	0.1455	0.1188	0.2883

Table S2. Computed Wiberg bond indices (WBI) and charges on optimized geometries of **1**, [**2**]$^{2-}$, **3** and [**4**]$^{2-}$ at the B3LYP/6-31G (d, p) level of theory.

	**1**	[**2**]$^{2-}$	**3**	[**4**]$^{2-}$
Wiberg BI				
C1-C2	0.74	0.06	0.65	0.06
C2-P1	0.82	1.23	0.80	1.24
P1-P1#	---	1.17	1.77	1.15
N1-C11	1.41	1.80	1.85	1.81
C1-C11	1.02	1.01	0.96	1.00
Charges				
$C_2B_{10}H_{10}$	-0.44	-0.88	-0.41	-0.89
P	0.26	0.00	0.22	0.00

(***Note***: the numbering system of selected atoms in this table is the same as the one in Figure S1-2.)

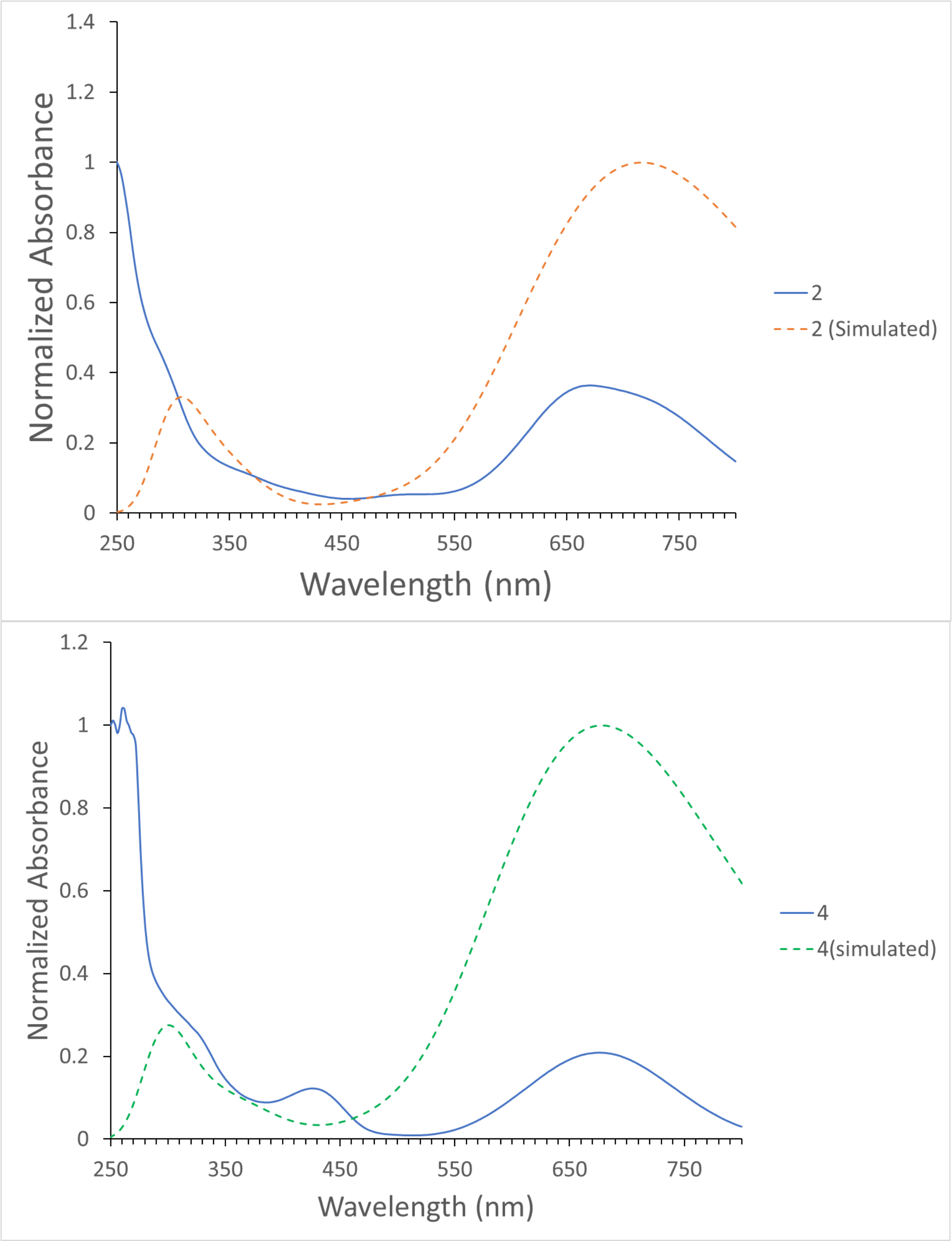

Figure S5. UV/Vis spectra of [**2**][K]$_2$ (up) and [**4**][CoCp$_2$]$_2$ (down) in CH_3CN.

The UV-Vis spectra of [**2**][K]$_2$ and [**4**][CoCp$_2$]$_2$ in CH_3CN were shown in Figure S5. The UV/Vis spectra of [**2**][K]$_2$ in CH_3CN displayed an intense absorption band at 671 nm for the delocalization system from the carborane moiety to another carborane moiety via the P-P bond. Time-dependent DFT (TD-DFT) calculations of [**2**]$^{2-}$ showed that the lowest energy absorption band is centered at 719 nm, which agreed with the experimental data. The absorption at 671 nm could be mainly assigned to the transition from the HOMO to the LUMO, corresponding to an electron transition from the π → π* orbital from the carborane moiety to another carborane moiety via the P-P bond. On the other hand, the UV/Vis spectra of [**4**][CoCp$_2$]$_2$ in CH_3CN displayed an intense absorption

band at 676 nm. Time-dependent DFT (TD-DFT) calculations of [**4**]$^{2-}$ showed that the lowest energy absorption band was centered at 667 nm, which agreed with the experimental data. The absorption band at 426 nm in [**4**][$CoCp_2$]$_2$ could be assigned to the transition from cobaltocenium.[6] The absorption band at 292 nm in [**4**][$CoCp_2$]$_2$ could be assignable to the $\pi \rightarrow \pi^*$ transition of the phenyl ring.

Computational Details. The geometry optimizations of compounds **1**, [**2**]$^{2-}$, **3**, and [**4**]$^{2-}$ were performed using the Gaussian09 program, Revision D.01,[7] at the B3LYP[8] level of theory using 6-31G(d,p) basis set. Frequency calculations were made to determine the characteristics of all stationary points as energy minima. Orbital energies of compounds [**2**]$^{2-}$ and [**4**]$^{2-}$ were calculated at the B3LYP/6-311++G(d,p) level of theory. NBO analysis at the B3LYP/6-31G(d,p) level of theory was carried out using the NBO program implemented in the Gaussian 09 package. The graphics of the molecular orbitals were produced by using the visualizing software VMD.[9]

Table S3. The Cartesian coordinates for **1**, [**2**]$^{2-}$, **3** and [**4**]$^{2-}$.

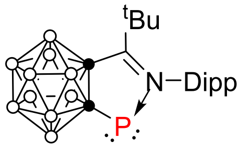

1

P	-0.21313600	-0.02685700	-1.89118200
N	0.39686300	-0.00319000	-0.24286900
C	-1.92448300	0.00344600	-1.21944800
C	-0.39687000	0.03739600	0.83725100
C	1.86605200	-0.07940600	-0.26861000
C	-1.84728100	0.00607300	0.42452000
B	-2.36601400	-1.46355200	-0.39177300
B	-2.38377600	1.46477900	-0.38176700
B	-3.25458100	-0.89238600	-1.80995200
B	-3.26607900	0.89572900	-1.80263300
C	0.03280100	0.29483300	2.30121100
C	2.46577400	-1.35622800	-0.25543300
C	2.61010100	1.10362900	-0.44800400
B	-3.19989600	-0.91767100	1.05835300
B	-3.20397300	0.87568400	1.07041400
H	-1.59822000	-2.35481200	-0.42589000
B	-4.11775900	-1.45453300	-0.36023300
H	-1.62262400	2.36319200	-0.40149000
B	-4.13391800	1.43065300	-0.34093600
H	-3.11010000	-1.47887300	-2.82399000
B	-4.68397500	-0.00921700	-1.24111200
H	-3.13148900	1.49477500	-2.81074700
C	-0.84954800	-0.50405300	3.29054400
C	-0.13404800	1.80936700	2.60946900
C	1.49107800	-0.08524300	2.63747100
C	3.85775000	-1.41270200	-0.36422400
C	1.67640100	-2.66155400	-0.17633000
C	3.99930700	0.97877300	-0.55505000
C	1.98732800	2.49349500	-0.55071400
H	-3.06781000	-1.53639700	2.04799200
B	-4.64229400	-0.02211100	0.55530200
H	-3.08223200	1.46286700	2.08305700
H	-4.68901800	-2.48943300	-0.34642900
H	-4.72050700	2.45667000	-0.31421600
H	-5.69181900	-0.01203600	-1.85887500
H	-0.46087200	-0.34734500	4.29972400
H	-0.81854200	-1.57579500	3.08234100
H	-1.88750700	-0.18303200	3.28799000
H	0.13133000	1.98729500	3.65599000
H	0.52488300	2.41584900	1.98866000
H	-1.15615000	2.15252100	2.46164900
H	1.63558100	0.09043300	3.70632800
H	1.69266100	-1.13868300	2.44761000
H	2.23076500	0.50853300	2.10714000
H	4.35076500	-2.37738400	-0.35168900
C	4.62106800	-0.26102200	-0.50431100
H	0.63752100	-2.41919300	0.04925500
C	2.18152200	-3.60376800	0.93306500
C	1.69380700	-3.39139100	-1.53549500
H	4.60136800	1.86956500	-0.68779800
H	0.92475500	2.40895900	-0.31775300
C	2.61236200	3.48869600	0.44686400
C	2.09455200	3.04505600	-1.98649600
H	-5.61320100	-0.02766200	1.23001800
H	5.69978900	-0.33144900	-0.58850500
H	2.19244000	-3.12227400	1.91332100
H	3.19292300	-3.96534800	0.72919900
H	1.53029900	-4.47978500	0.99786200
H	1.29752800	-2.75718300	-2.33008000
H	1.08094200	-4.29604400	-1.48519700
H	2.71067400	-3.68986700	-1.80757400
H	2.60461200	3.10693800	1.47032000
H	3.64921800	3.71969100	0.18842200
H	2.05623000	4.43017800	0.43225500
H	1.60757500	2.38050200	-2.70190800
H	1.61251400	4.02498000	-2.04985400
H	3.14002400	3.16556400	-2.28532700

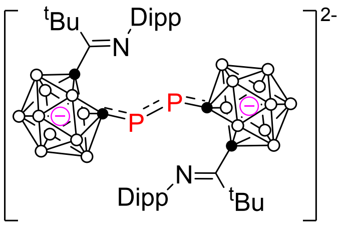

[**2**]$^{2-}$

P	-0.14619600	-0.75391000	-0.77639500
N	-5.03256500	-0.08150100	0.23018900
C	-3.59198600	-1.89573800	-0.16577300

C	-1.14771100	-1.93635600	0.03429400	H	-3.88119700	3.77993300	2.31060300
C	-4.46384900	-0.76325300	-0.69188000	H	-3.66509500	2.03672700	2.56265500
C	-4.58246200	-0.48746700	-2.22769300	B	-2.60812000	-1.57025000	1.18075900
C	-5.68840300	0.53034100	-2.57854400	H	-2.61874100	-0.47745200	1.63284600
H	-6.68024100	0.19301700	-2.26667400	B	-4.10255200	-2.63890800	1.24004800
H	-5.51581400	1.51345400	-2.14018500	H	-5.12098500	-2.27495300	1.72311900
H	-5.70567600	0.65108700	-3.66832400	B	-3.99852600	-3.49955900	-0.30518600
C	-4.92001100	-1.77438300	-3.01762200	H	-4.93652600	-3.79920300	-0.97095200
H	-5.87752800	-2.19525200	-2.69336000	B	-2.41928900	-2.78317000	-1.00000900
H	-5.00928000	-1.51741600	-4.08038500	H	-2.26753000	-2.65601100	-2.16801700
H	-4.16132100	-2.54725400	-2.92395900	B	-1.13850100	-2.64002900	1.49540600
C	-3.24342300	0.10658100	-2.72287200	H	-0.23609500	-2.33886300	2.20790500
H	-2.38565900	-0.52967100	-2.50716500	B	-2.69661000	-3.05848200	2.20783200
H	-3.29143600	0.25461100	-3.80891000	H	-2.77002300	-3.04725800	3.39737500
H	-3.05252700	1.08060200	-2.26239400	B	-3.51306900	-4.30276800	1.20065600
C	-5.91362400	0.98907400	0.29624800	H	-4.17487400	-5.18899300	1.64816000
C	-7.31204300	0.74204700	0.40957000	B	-2.52012700	-4.45775800	-0.28370300
C	-8.17353300	1.82167800	0.61813000	H	-2.45723100	-5.46380500	-0.92127300
H	-9.24199800	1.63505300	0.69581700	B	-1.01806100	-3.55114100	-0.08707400
C	-7.69898100	3.12617300	0.73590300	H	-0.02935600	-3.96207100	-0.60499500
H	-8.38746000	3.95260900	0.89337600	B	-1.76558400	-4.31893000	1.33687800
C	-6.32611600	3.35149100	0.66453900	H	-1.19501900	-5.21566600	1.87874800
H	-5.94495400	4.36329500	0.77805200	P	0.14631500	0.75342800	0.77774800
C	-5.41463300	2.31214600	0.45853400	N	5.03248200	0.08159300	-0.23051900
C	-7.85946500	-0.68240900	0.37520700	C	3.59188100	1.89562100	0.16635800
H	-7.08752700	-1.32628700	-0.05415600	C	1.14757000	1.93607200	-0.03297700
C	-9.11789800	-0.83714100	-0.49704800	C	4.46397000	0.76305300	0.69189000
H	-9.40850200	-1.89241400	-0.55859400	C	4.58305000	0.48686600	2.22759000
H	-9.97377900	-0.28907200	-0.08568000	C	5.68915700	-0.53097100	2.57783900
H	-8.94909800	-0.47257800	-1.51579400	H	6.68087300	-0.19353200	2.26571200
C	-8.12163200	-1.19477100	1.80554100	H	5.51647100	-1.51399400	2.13931300
H	-8.46585700	-2.23603900	1.79169600	H	5.70680300	-0.65196700	3.66758500
H	-7.20819200	-1.14980800	2.40360300	C	4.92072300	1.77359300	3.01776800
H	-8.88835000	-0.58732000	2.30246000	H	5.87817200	2.19455700	2.69343000
C	-3.91599900	2.59807600	0.46718800	H	5.01019300	1.51637700	4.08045500
H	-3.39750900	1.69868700	0.12769500	H	4.16200000	2.54647100	2.92442600
C	-3.51145500	3.74235900	-0.47958400	C	3.24418100	-0.10739600	2.72298000
H	-2.42266300	3.85165000	-0.49540900	H	2.38635800	0.52900700	2.50795400
H	-3.84865800	3.55395300	-1.50475200	H	3.29261500	-0.25600600	3.80892100
H	-3.93399600	4.70357900	-0.16284500	H	3.05306800	-1.08116200	2.26205500
C	-3.41984800	2.87229500	1.90069000	C	5.91367500	-0.98883100	-0.29712900
H	-2.33300300	3.00149900	1.90872000	C	7.31200900	-0.74155200	-0.41097200

C	8.17359300	-1.82099600	-0.62010200
H	9.24199400	-1.63417200	-0.69819100
C	7.69920900	-3.12554500	-0.73795300
H	8.38775800	-3.95183500	-0.89588300
C	6.32641100	-3.35110300	-0.66606800
H	5.94536800	-4.36294600	-0.77963400
C	5.41483800	-2.31195400	-0.45946500
C	7.85922200	0.68298300	-0.37654100
H	7.08732100	1.32667400	0.05316900
C	9.11790200	0.83775300	0.49535300
H	9.40839900	1.89305400	0.55694900
H	9.97372300	0.28983800	0.08365300
H	8.94945800	0.47303900	1.51410300
C	8.12087000	1.19563200	-1.80686600
H	8.46493600	2.23695000	-1.79294700
H	7.20725000	1.15063000	-2.40465200
H	8.88752800	0.58838900	-2.30413200
C	3.91625000	-2.59814800	-0.46751800
H	3.39773600	-1.69890100	-0.12767300
C	3.51234400	-3.74264000	0.47927700
H	2.42358200	-3.85219200	0.49552600
H	3.84991600	-3.55427900	1.50433200
H	3.93499000	-4.70372100	0.16225300
C	3.41952400	-2.87227100	-1.90083800
H	2.33269300	-3.00161900	-1.90841500
H	3.88081900	-3.77979700	-2.31105900
H	3.66436700	-2.03659200	-2.56281200
B	2.60762400	1.57041100	-1.17996400
H	2.61823100	0.47774400	-1.63237200
B	4.10197600	2.63921100	-1.23941700
H	5.12028900	2.27544100	-1.72287900
B	3.99835200	3.49943800	0.30608700
H	4.93653600	3.79896000	0.97165000
B	2.41939600	2.78273700	1.00120100
H	2.26800300	2.65522800	2.16921900
B	1.13785300	2.64016900	-1.49389100
H	0.23525300	2.33913600	-2.20620200
B	2.69570400	3.05894800	-2.20665600
H	2.76876800	3.04805500	-3.39622400
B	3.51237400	4.30301700	-1.19938800
H	4.17397100	5.18941500	-1.64685500
B	2.51986900	4.45751800	0.28531800
H	2.45706800	5.46338800	0.92317600
B	1.01782300	3.55081100	0.08889500
H	0.02924100	3.96151700	0.60722600
B	1.76484400	4.31907700	-1.33507100
H	1.19403500	5.21591500	-1.87651300

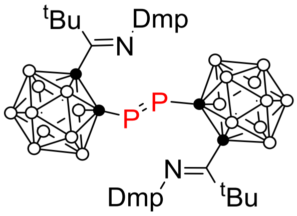

3

P	-0.74623800	-0.08244900	0.70419200
N	-3.20088700	0.59346100	-0.10417600
C	-3.27937400	-1.75577000	-0.07756700
C	-1.55868500	-1.70541400	0.15005900
C	-4.00843900	-0.38240800	0.00830800
C	-5.55212900	-0.31247100	0.26083600
C	-6.11629400	1.10149600	-0.00700300
H	-5.72201000	1.85752600	0.66982200
H	-5.93359100	1.43239900	-1.03193000
H	-7.20009300	1.06069200	0.13880500
C	-6.36504100	-1.26769200	-0.64597700
H	-6.16857400	-2.32067500	-0.46282100
H	-7.42772200	-1.09409400	-0.45049500
H	-6.18676900	-1.06078000	-1.70515500
C	-5.82723800	-0.64656600	1.75112200
H	-5.52625000	-1.65677200	2.02454500
H	-5.31679400	0.05466100	2.41539600
H	-6.90329600	-0.55819100	1.93375800
C	-3.35384500	1.99894900	-0.14655100
C	-3.36090100	2.61250500	-1.42085000
C	-3.38133900	4.00716000	-1.48812300
H	-3.40010300	4.48173200	-2.46537900
C	-3.36056700	4.78882600	-0.33486700
H	-3.36298300	5.87185400	-0.40637000
C	-3.31603600	4.16724000	0.90837900
H	-3.28203700	4.76821600	1.81314600
C	-3.30581900	2.77251800	1.03281700
C	-3.27680600	2.15287800	2.40969500
H	-2.71535300	1.21648700	2.43025300
H	-2.81181500	2.83668500	3.12467500

H	-4.28860300	1.94326500	2.77960400
C	-3.34777700	1.78646300	-2.68317900
H	-2.46898400	1.13477000	-2.72245800
H	-4.22664500	1.13550700	-2.76489300
H	-3.33180000	2.43220100	-3.56450600
B	-2.58233600	-2.38791900	1.37169600
H	-2.74379300	-1.77492000	2.36781400
B	-3.87557800	-3.25076400	0.50502400
H	-4.94935400	-3.29260000	0.98616300
B	-3.63603100	-2.97392700	-1.23547600
H	-4.53239400	-2.79269400	-1.97895600
B	-2.18712700	-1.95727800	-1.42637700
H	-2.11642800	-1.08380100	-2.21191500
B	-1.03620300	-3.14107300	0.91569900
H	-0.13643900	-3.04190800	1.67610000
B	-2.48491200	-4.14119000	1.13578300
H	-2.60220300	-4.86729200	2.06529700
B	-3.13478700	-4.50997500	-0.49060900
H	-3.72930900	-5.50885000	-0.72453100
B	-2.09258000	-3.70702000	-1.69628700
H	-1.92950500	-4.12162600	-2.79497000
B	-0.79833600	-2.88815800	-0.82176000
H	0.26863400	-2.63318400	-1.25391600
B	-1.38204300	-4.45349000	-0.23357400
H	-0.68682400	-5.41223400	-0.28248100
P	0.74635700	0.08237600	-0.70415800
N	3.20093300	-0.59350300	0.10417500
C	3.27943900	1.75572400	0.07774000
C	1.55877200	1.70537100	-0.15006400
C	4.00853000	0.38237400	-0.00795900
C	5.55226800	0.31260500	-0.26026700
C	6.11668500	-1.10123700	0.00769400
H	5.72275900	-1.85735100	-0.66924100
H	5.93381100	-1.43219600	1.03257000
H	7.20051200	-1.06017400	-0.13784300
C	6.36486300	1.26802500	0.64661500
H	6.16819100	2.32096200	0.46339200
H	7.42760600	1.09464500	0.45127800
H	6.18649300	1.06111700	1.70577700
C	5.82752600	0.64673800	-1.75051600
H	5.52640500	1.65689600	-2.02397700
H	5.31728500	-0.05457300	-2.41486000
H	6.90362300	0.55854200	-1.93300800
C	3.35375000	-1.99901400	0.14634600
C	3.36047900	-2.61278800	1.42054100
C	3.38073800	-4.00745700	1.48758200
H	3.39925900	-4.48219700	2.46476100
C	3.36009200	-4.78892600	0.33419000
H	3.36236300	-5.87196600	0.40551000
C	3.31587000	-4.16712300	-0.90895900
H	3.28197300	-4.76794200	-1.81383400
C	3.30584400	-2.77238000	-1.03316200
C	3.27718400	-2.15249500	-2.40993700
H	2.71572100	-1.21610900	-2.43047500
H	2.81239800	-2.83618300	-3.12516300
H	4.28907500	-1.94279400	-2.77954000
C	3.34720500	-1.78696400	2.68301100
H	2.46846000	-1.13520500	2.72225200
H	4.22611300	-1.13609500	2.76499100
H	3.33102200	-2.43285400	3.56422300
B	2.58256300	2.38776800	-1.37164500
H	2.74411600	1.77469200	-2.36770000
B	3.87572600	3.25065900	-0.50490100
H	4.94955800	3.29243600	-0.98592700
B	3.63599200	2.97396000	1.23559300
H	4.53227700	2.79276900	1.97918000
B	2.18705100	1.95734600	1.42641800
H	2.11625600	1.08393100	2.21201500
B	1.03639200	3.14098000	-0.91586700
H	0.13670700	3.04177100	-1.67635600
B	2.48514000	4.14105900	-1.13587500
H	2.60254100	4.86708900	-2.06543300
B	3.13485000	4.50995800	0.49055700
H	3.72936400	5.50884100	0.72446400
B	2.09250200	3.70711000	1.69618700
H	1.92931800	4.12180200	2.79482100
B	0.79833800	2.88820100	0.82158600
H	-0.26868200	2.63327700	1.25364900
B	1.38213100	4.45348100	0.23334100
H	0.68692100	5.41223900	0.28210200

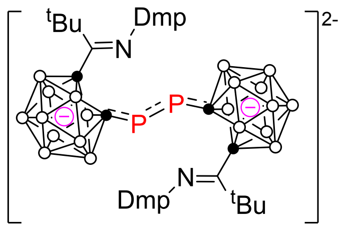

[4]$^{2-}$

P	0.61429400	0.06995500	0.91038600
N	4.39018600	-0.27722800	0.35277000
C	3.64523500	1.91780500	-0.05936700
C	1.33696200	1.65152400	0.74082900
C	4.48296500	0.71238200	-0.45012700
C	5.39583300	0.76367600	-1.72642100
C	6.51272500	1.81728900	-1.51587800
H	6.12605600	2.81498200	-1.32097700
H	7.15787000	1.53264000	-0.67763900
H	7.13853300	1.86283500	-2.41641800
C	4.56104100	1.09862200	-2.98544100
H	4.06203300	2.06262000	-2.92358200
H	5.22500500	1.11412600	-3.85906500
H	3.79680900	0.33428700	-3.15778700
C	6.11393300	-0.57008600	-2.02473400
H	5.41735800	-1.38776400	-2.21556400
H	6.71907000	-0.43366400	-2.92956000
H	6.78444300	-0.87946100	-1.22134400
C	4.98500100	-1.52450600	0.47065300
C	6.10991000	-1.66509800	1.32390700
C	6.63569000	-2.93946100	1.54949000
H	7.50508200	-3.03620600	2.19779500
C	6.06175000	-4.07446400	0.97891400
H	6.47783300	-5.06011500	1.17098700
C	4.92868700	-3.92801700	0.17850700
H	4.45281500	-4.80570800	-0.25340100
C	4.36717900	-2.67583400	-0.08180700
C	3.12576100	-2.54321100	-0.92346300
H	2.35983000	-1.96140700	-0.40195800
H	3.31867400	-2.02133100	-1.86961200
H	2.70564000	-3.52321100	-1.16320500
C	6.71222100	-0.44908800	1.98173700
H	5.93797900	0.15534200	2.46585600
H	7.45484300	-0.73921300	2.73235200
H	7.21023000	0.21231900	1.26083900
B	2.95844700	1.89600100	1.51837600
H	3.29850800	1.05437200	2.27600400
B	4.23141000	3.11479200	0.91648700
H	5.37371000	3.05469600	1.24824400
B	3.66840300	3.43560100	-0.73807100
H	4.36344100	3.68438000	-1.66467500
B	2.20858900	2.30956800	-0.83241800
H	1.91230300	1.74354900	-1.83042600
B	1.42160000	2.82858700	1.86788000
H	0.74271500	2.67099400	2.83027100
B	2.96718500	3.65301800	2.02323200
H	3.27035400	4.03859100	3.11070600
B	3.31125300	4.59169200	0.54278000
H	3.83999500	5.66181500	0.54397000
B	2.04848500	4.10102100	-0.64670000
H	1.65900300	4.82503900	-1.50979200
B	0.84485500	3.07791900	0.15166000
H	-0.27979900	3.14264000	-0.22135600
B	1.64887900	4.40383100	1.07173200
H	1.01042600	5.34174300	1.44126000
P	-0.61391300	-0.07006300	-0.90905000
N	-4.39005400	0.27712800	-0.35330300
C	-3.64527600	-1.91776600	0.06001400
C	-1.33652500	-1.65161100	-0.73938200
C	-4.48331500	-0.71224100	0.44982800
C	-5.39709000	-0.76320500	1.72549500
C	-6.51375500	-1.81697000	1.51450300
H	-6.12689200	-2.81468200	1.32010500
H	-7.15838200	-1.53258900	0.67577200
H	-7.14014000	-1.86231600	2.41465000
C	-4.56315600	-1.09765000	2.98521600
H	-4.06393200	-2.06157500	2.92398900
H	-5.22775300	-1.11302700	3.85836300
H	-3.79919500	-0.33311900	3.15789300
C	-6.11553000	0.57058200	2.02287000
H	-5.41916200	1.38839100	2.21387800
H	-6.72125400	0.43440800	2.92734000
H	-6.78553300	0.87961500	1.21892700
C	-4.98473800	1.52439500	-0.47187400
C	-6.10905300	1.66480700	-1.32594500
C	-6.63465000	2.93912000	-1.55221100
H	-7.50359000	3.03571900	-2.20114200
C	-6.06108500	4.07425400	-0.98151300

H	-6.47700900	5.05986900	-1.17411700
C	-4.92858800	3.92797900	-0.18027700
H	-4.45299900	4.80576900	0.25174300
C	-4.36728700	2.67584900	0.08074000
C	-3.12647700	2.54342800	0.92333300
H	-2.36037800	1.96102600	0.40274900
H	-3.32019500	2.02232400	1.86975300
H	-2.70621400	3.52347200	1.16264700
C	-6.71093500	0.44863200	-1.98386300
H	-5.93639100	-0.15585000	-2.46743600
H	-7.45316400	0.73856300	-2.73494000
H	-7.20928900	-0.21266500	-1.26310400
B	-2.95745400	-1.89647100	-1.51740800
H	-3.29731500	-1.05521100	-2.27554600
B	-4.23083600	-3.11503800	-0.91576100
H	-5.37292000	-3.05510600	-1.24828700
B	-3.66889000	-3.43539100	0.73925600
H	-4.36456500	-3.68387400	1.66546100
B	-2.20928100	-2.30926700	0.83412400
H	-1.91346600	-1.74271500	1.83197200
B	-1.42051700	-2.82931900	-1.86584000
H	-0.74108700	-2.67219400	-2.82792200
B	-2.96600600	-3.65376000	-2.02160100
H	-3.26866000	-4.03978100	-3.10906200
B	-3.31089600	-4.59184200	-0.54100000
H	-3.83958300	-5.66199300	-0.54212300
B	-2.04882800	-4.10061700	0.64904300
H	-1.65963200	-4.82427300	1.51256700
B	-0.84485500	-3.07762000	-0.14907000
H	0.27958000	-3.14230100	0.22464200
B	-1.64821800	-4.40412400	-1.06903600
H	-1.00950900	-5.34218800	-1.4377290

References

(1) Chan, T. L.; Xie, Z. The synthesis, structure and reactivity of an imine-stabilized carboranylphosphorus(I) compound. *Chem. Commun.* **2016**, *52*, 7280-7283.

(2) Chan, T. L.; Zhang, J.; Xie, Z. Carboranyl diphosphenes: synthesis, structure and reactivity. *Dalton Trans*. **2025**, *54*, 10946–10955.

(3) Wang, H.; Zhang, J.; Lin, Z.; Xie, Z. The synthesis and structure of a carbene-stabilized iminocarboranyl-boron(I) compound. *Chem. Commun.* **2015**, *51*, 16817-16820.

(4) Sheldrick, G. M. SADABS: Program for Empirical Absorption Correction of Area Detector Data. University of Göttingen: Germany, 1996.

(5) Sheldrick, G. M. SHELXTL 5.10 for Windows NT: Structure Determination Software Programs. Bruker Analytical X-ray systems, Inc.: Madison, Wisconsin, USA, 1997.

(6) Warratz, R.; Peters, G.; Studt, F.; Römer, R.-H.; Tuczek, F. Orbital Interactions in Fe(Ii)/Co(Iii) Heterobimetallocenes: Single versus Double Bridge. *Inorg. Chem.* **2006**, *45*, 2531-2542.

(7) Scalmani, G.; Barone, V.; Mennucci, B.; Petersson, G. A.; Nakatsuji, H.; Caricato, M.; Li, X.; Hratchian, H. P.; Izmaylov, A. F.; Bloino, J.; Zheng, G.; Sonnenberg, J. L.; Hada, M.; Ehara, M.; Toyota, K.; Fukuda, R.; Hasegawa, J.; Ishida, M.; Nakajima, T.; Honda, Y.; Kitao, O.; Nakai, H.; Vreven, T.; Montgomery Jr., J. A.; Peralta, J. E.; Ogliaro, F.; Bearpark, M.; Heyd, J. J.; Brothers, E.; Kudin, K. N.; Staroverov, V. N.; Kobayashi, R.; Normand, J.; Raghavachari, K.; Rendell, A.; Burant, J. C.; Iyengar, S. S.; Tomasi, J.; Cossi, M.; Rega, N.; Millam, J. M.; Klene, M.; Knox, J. E.; Cross, J. B.; Bakken, V.; Adamo, C.; Jaramillo, J.; Gomperts, R.; Stratmann, R. E.; Yazyev, O.; Austin, A. J.; Cammi, R.; Pomelli, C.; Ochterski, J. W.; Martin, R. L.; Morokuma, K.; Zakrzewski, V. G.; Voth, G. A.; Salvador, P.; Dannenberg, J. J.; Dapprich, S.; Daniels, A. D.; Farkas, Ö.; Foresman, J. B.; Ortiz, J. V.; Cioslowski, J.; Fox, D. J. Gaussian 09, Revision D.01, Gaussian, Inc., Wallingford CT, **2009**.

(8) (a) Becke, A. D. Density-functional thermochemistry. III. The role of exact exchange. *J. Chem. Phys*. **1993**, *98*, 5648-5652. (b) Lee, C.; Yang, W.; Parr, R. G. Development of the Colle-Salvetti correlation-energy formula into a functional of the electron density. *Phys. Rev. B,* **1988**, *37*, 785-789.

(9) Humphrey, W.; Dalke, A.; Schulten, K. VMD: Visual molecular dynamics. *J. Mol. Graphics* **1996**, *14*, 33-38.

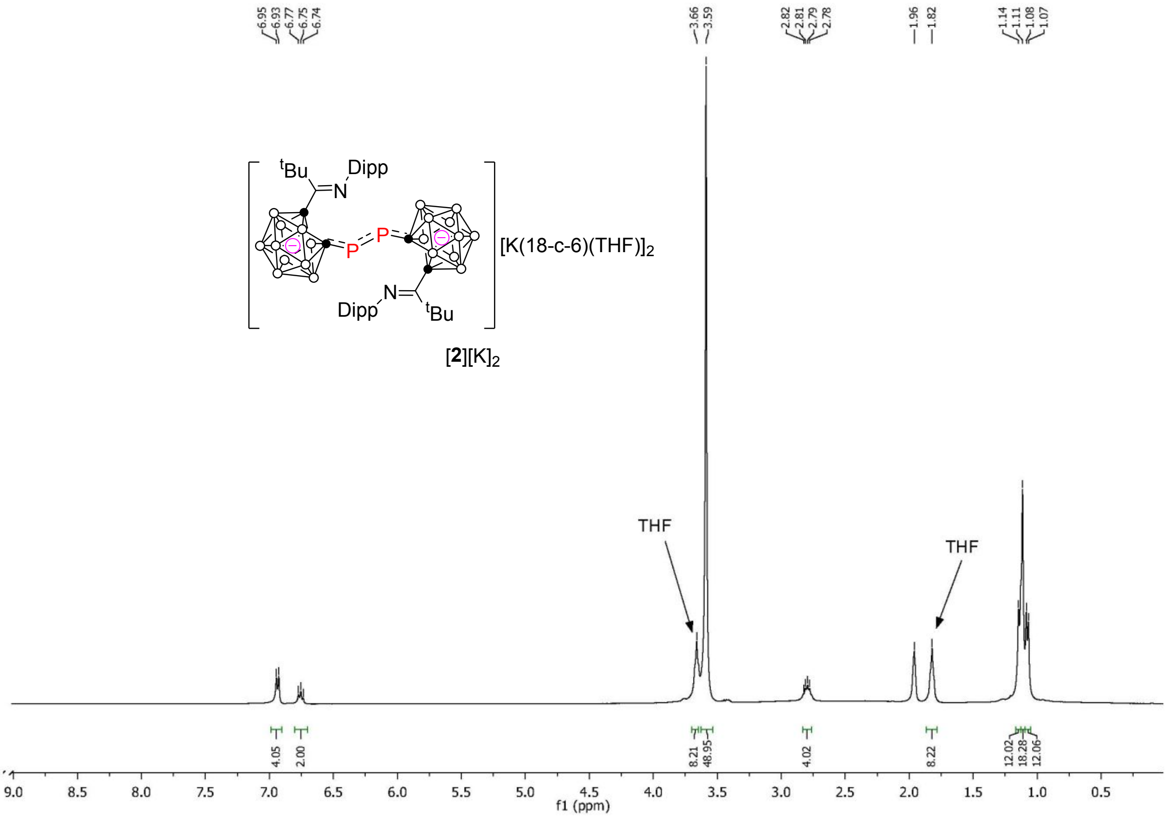

Figure S6. ^{1}H NMR spectrum of [**2**][K]$_2$ in CD_3CN.

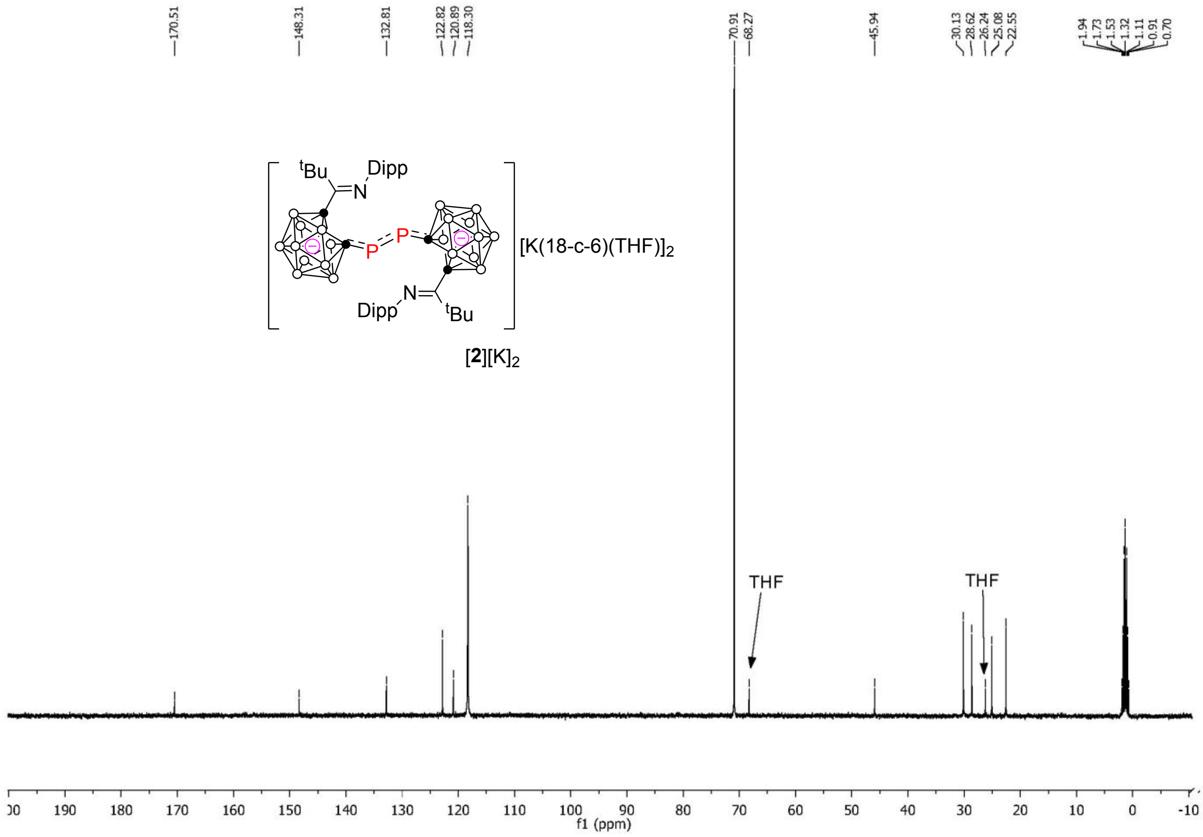

Figure S7. $^{13}C\{^{1}H\}$ NMR spectrum of [**2**][K]$_2$ in CD_3CN.

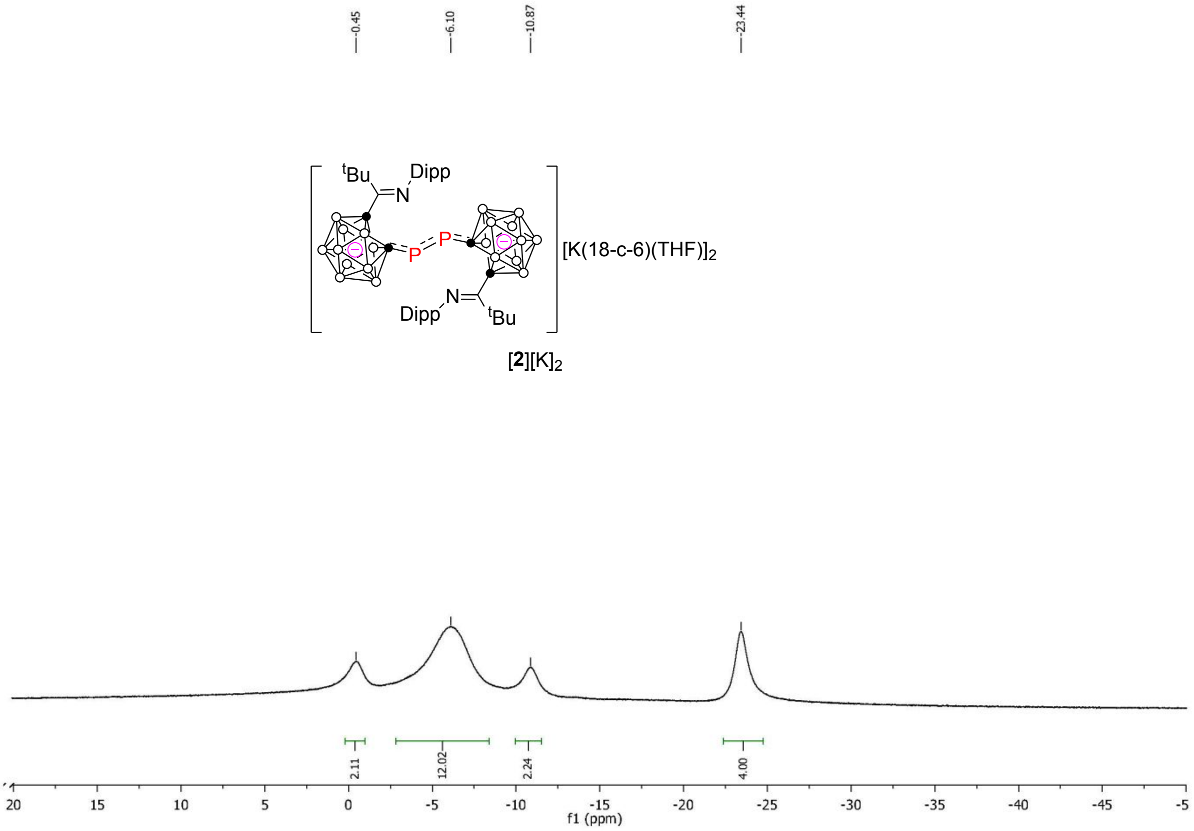

Figure S8. $^{11}B\{^{1}H\}$ NMR spectrum of [**2**][K]$_2$ in CD_3CN.

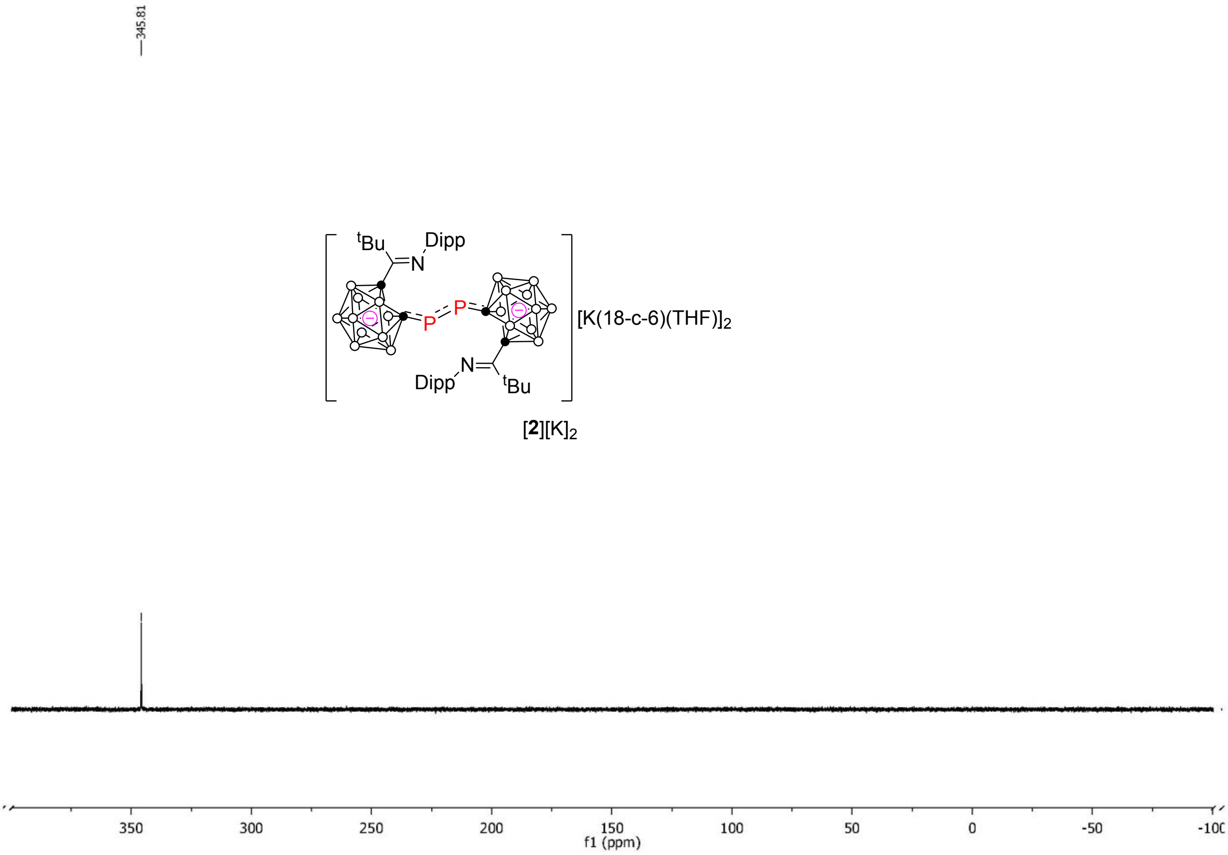

Figure S9. $^{31}P\{^{1}H\}$ NMR spectrum of [**2**][K]$_2$ in CD_3CN.

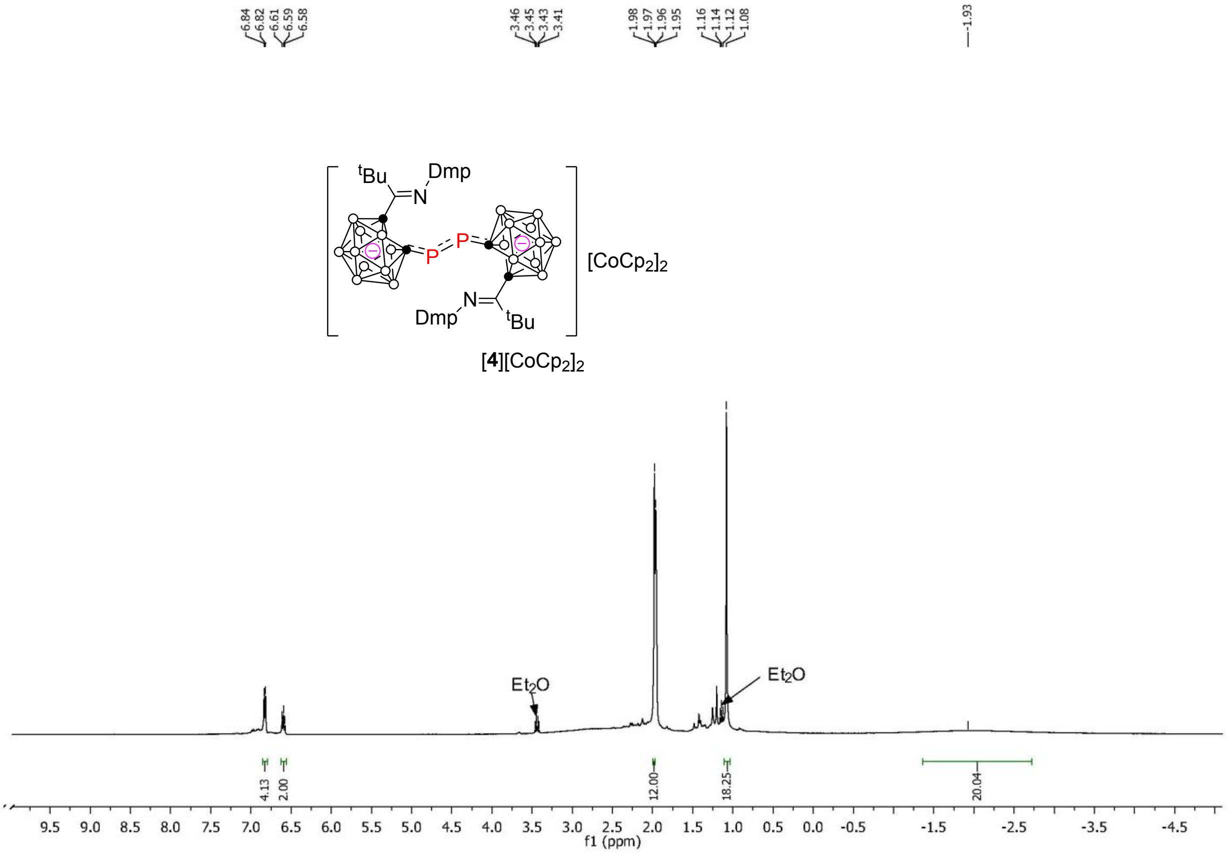

Figure S10. ^{1}H NMR spectrum of [**4**][$CoCp_2$]$_2$ in CD_3CN.

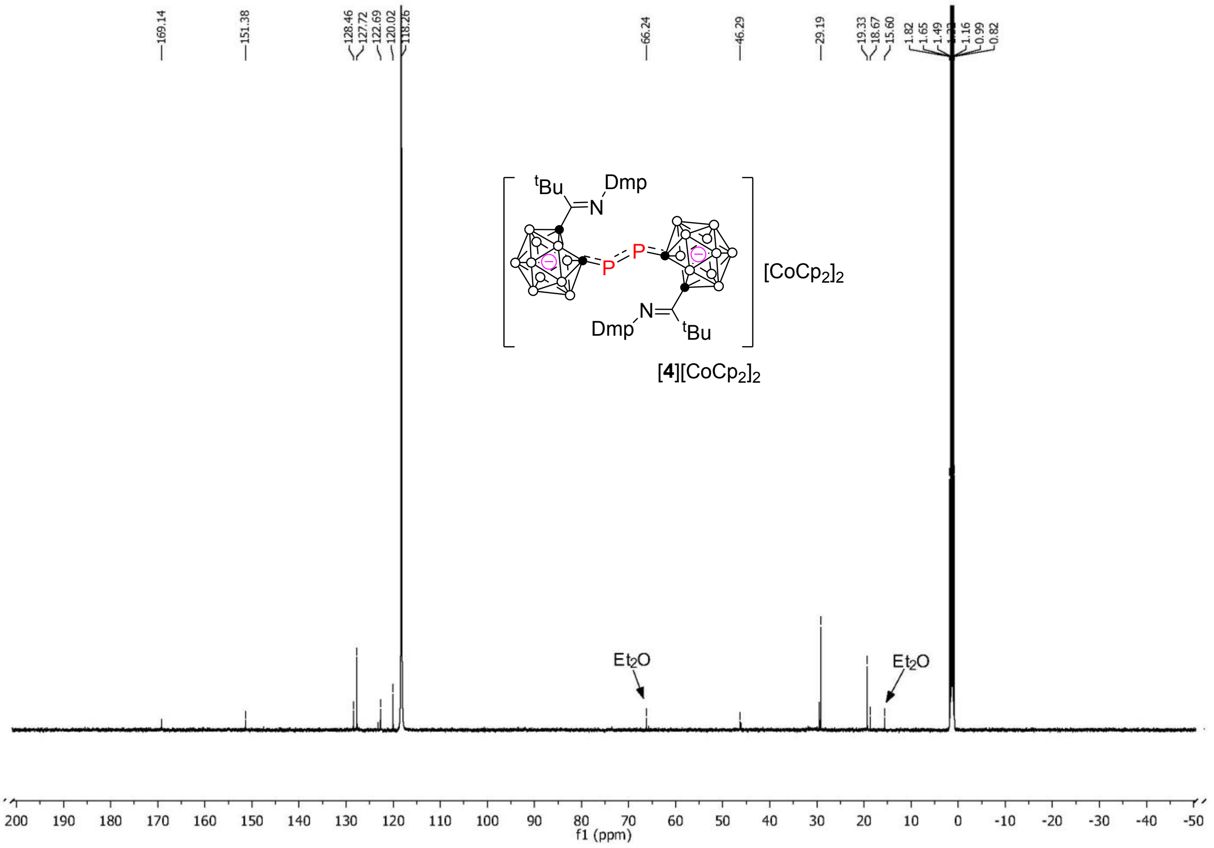

Figure S11. $^{13}C\{^1H\}$ NMR spectrum of [**4**][$CoCp_2$]$_2$ in CD_3CN.

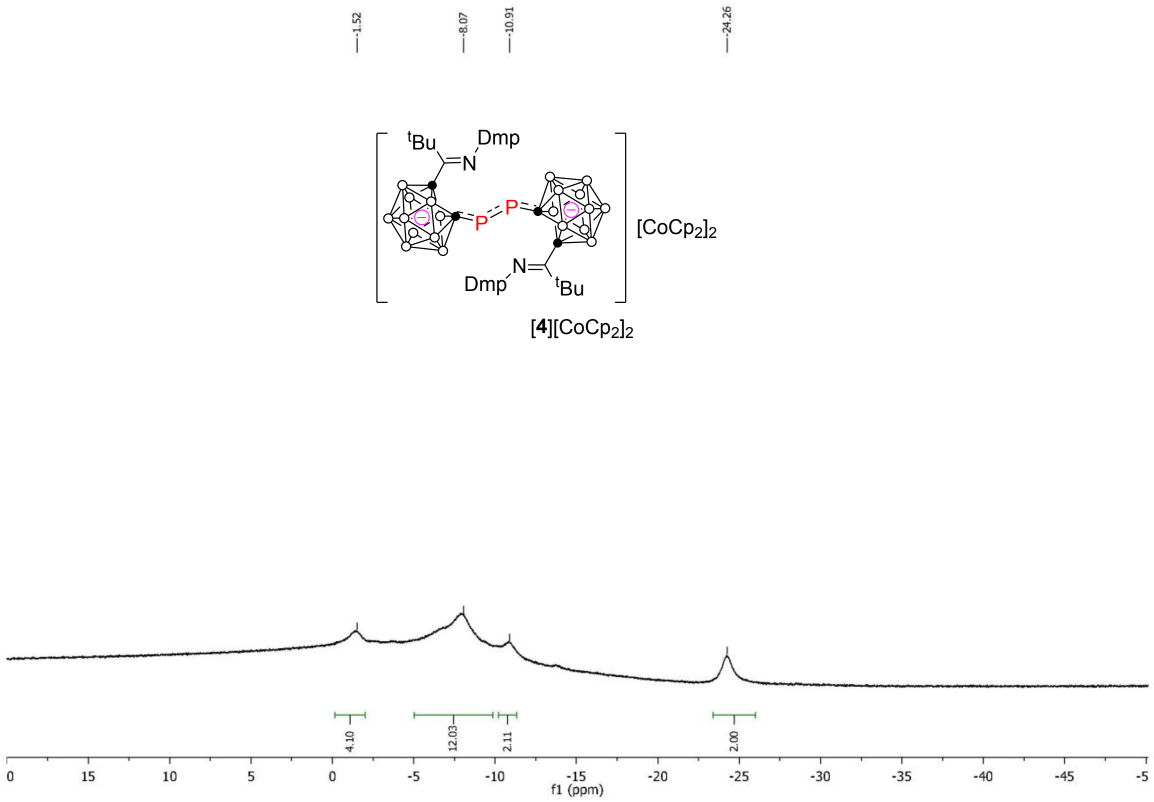

Figure S12. $^{11}B\{^1H\}$ NMR spectrum of [**4**]$[CoCp_2]_2$ in CD_3CN.

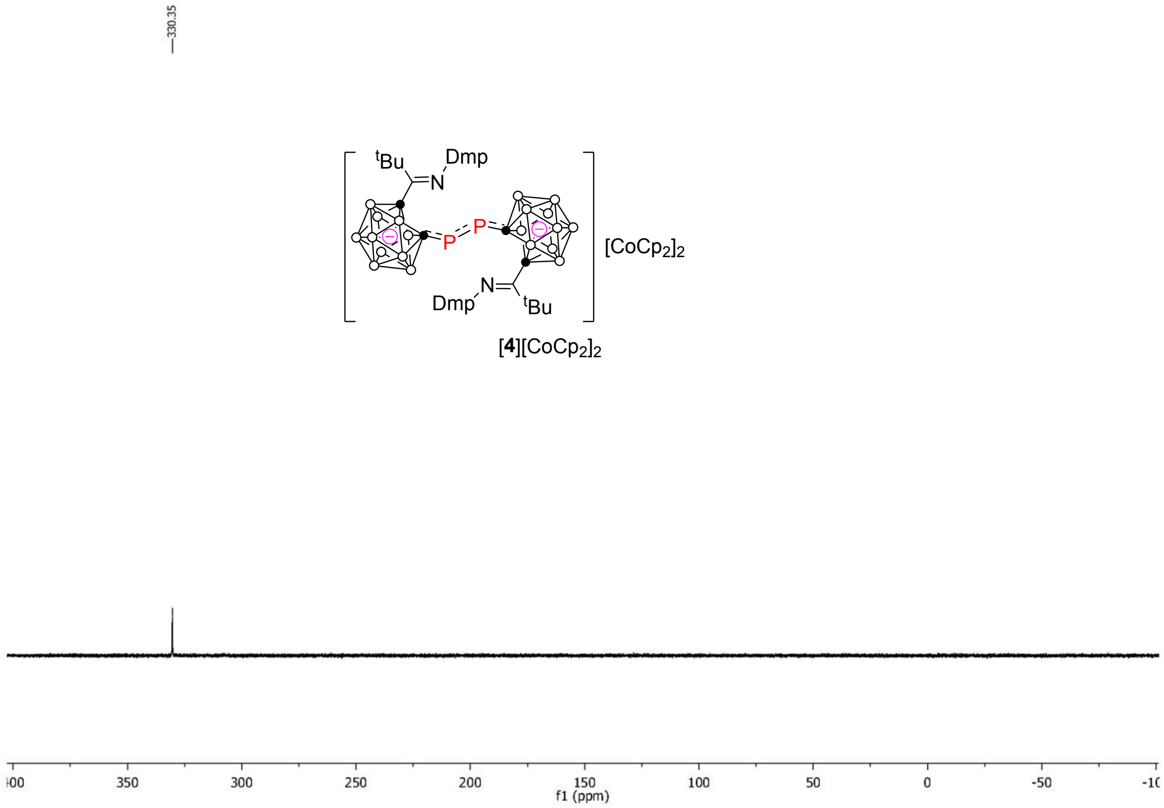

Figure S13. $^{31}P\{^1H\}$ NMR spectrum of [**4**]$[CoCp_2]_2$ in CD_3CN.

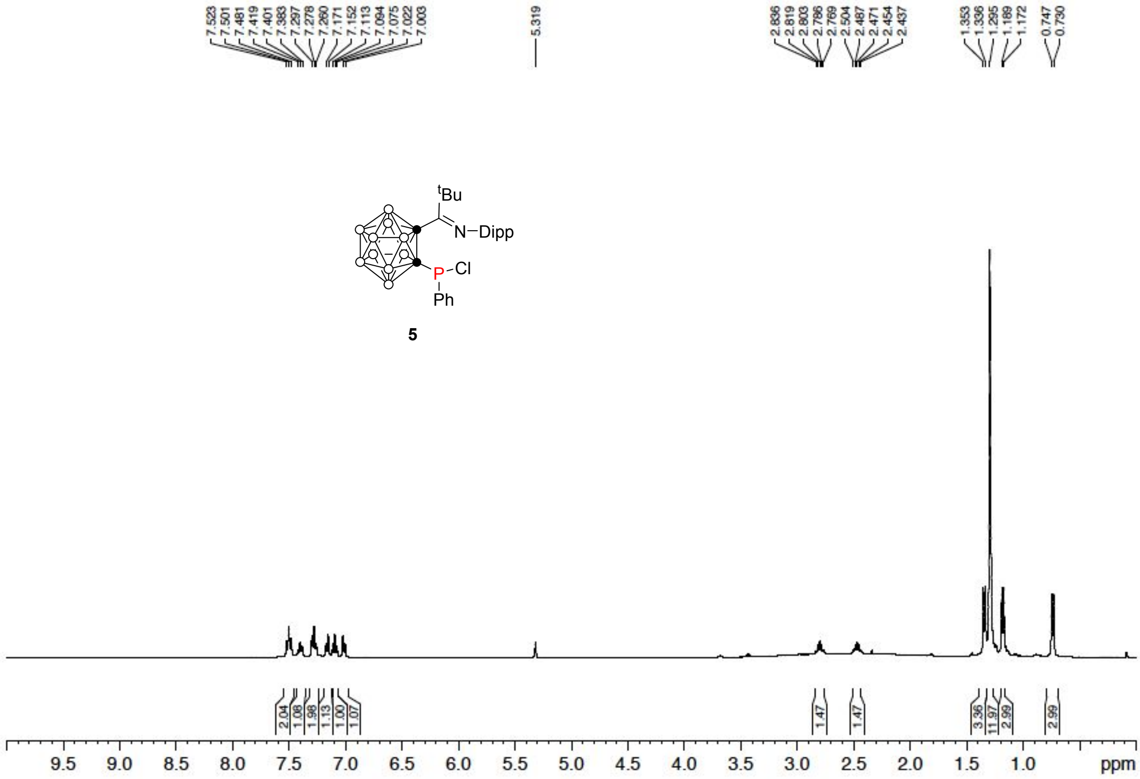

Figure S14. ^{1}H NMR spectrum of **5** in CD_2Cl_2.

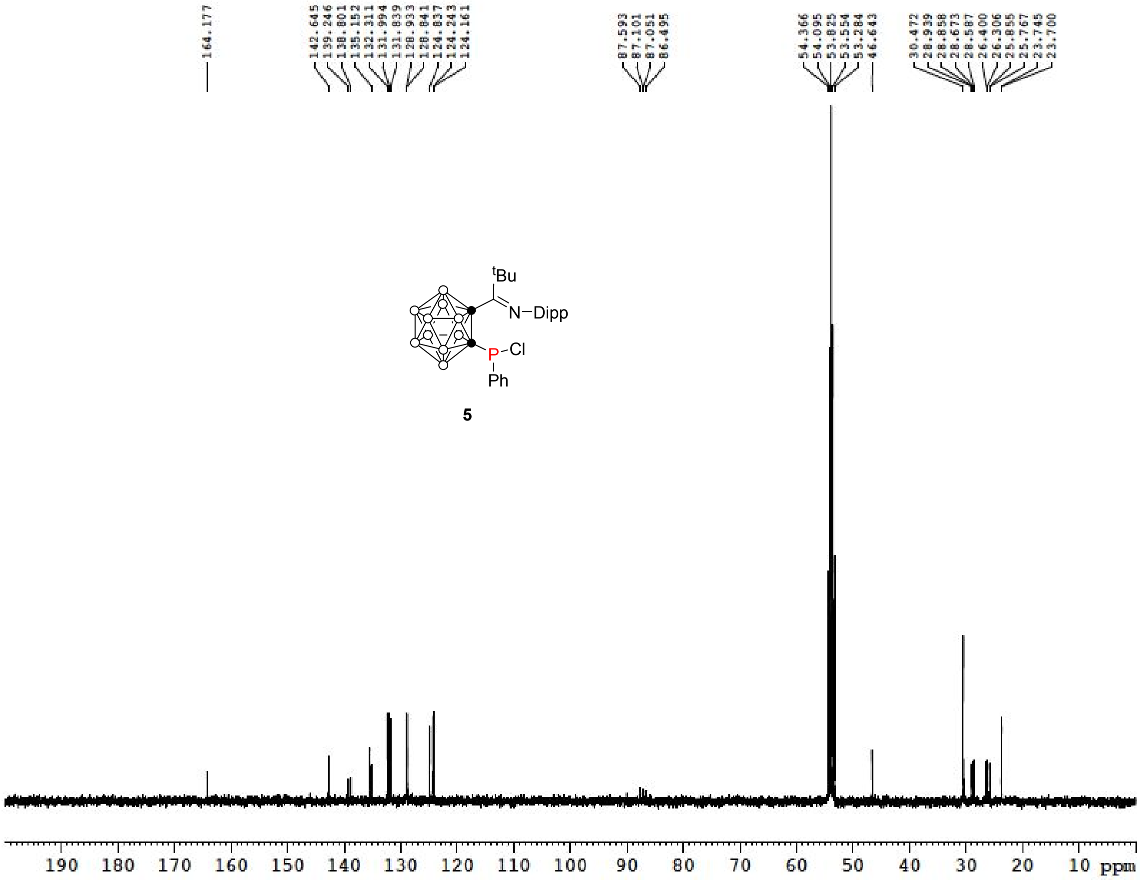

Figure S15. $^{13}C\{^{1}H\}$ NMR spectrum of **5** in CD_2Cl_2.

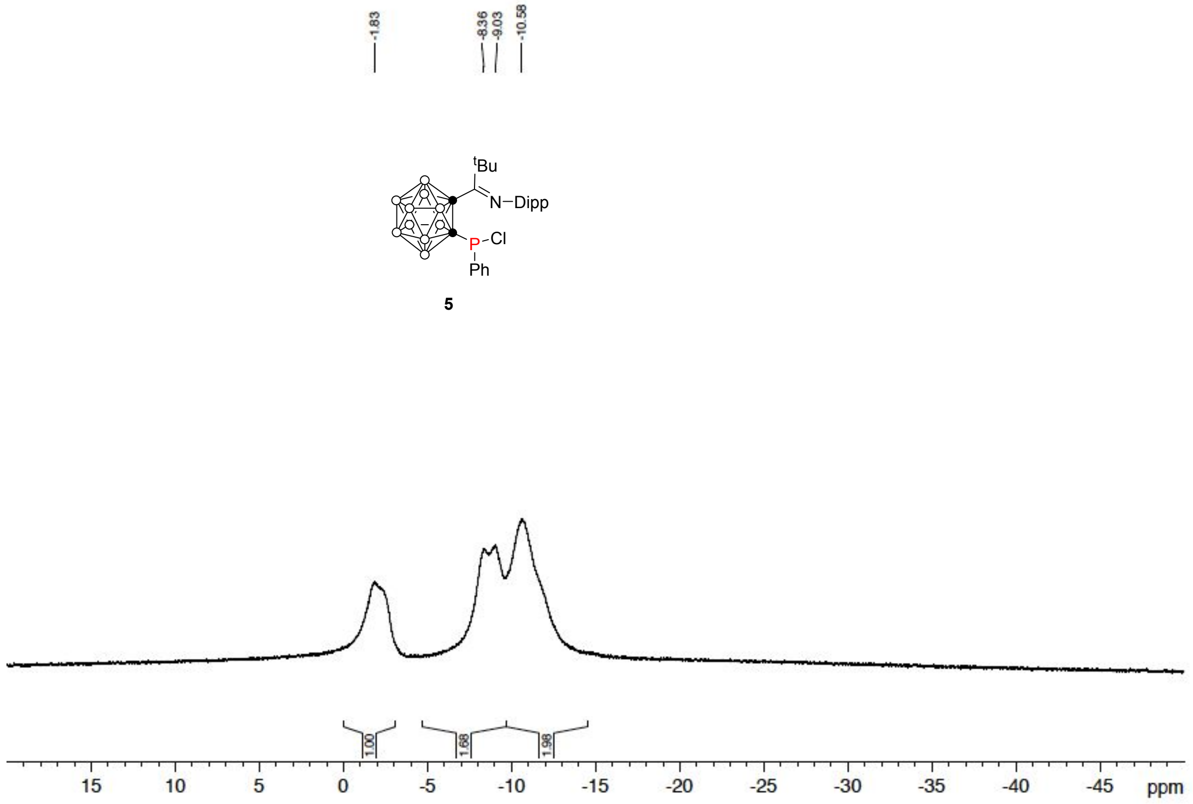

Figure S16. $^{11}B\{^{1}H\}$ NMR spectrum of **5** in CD_2Cl_2.

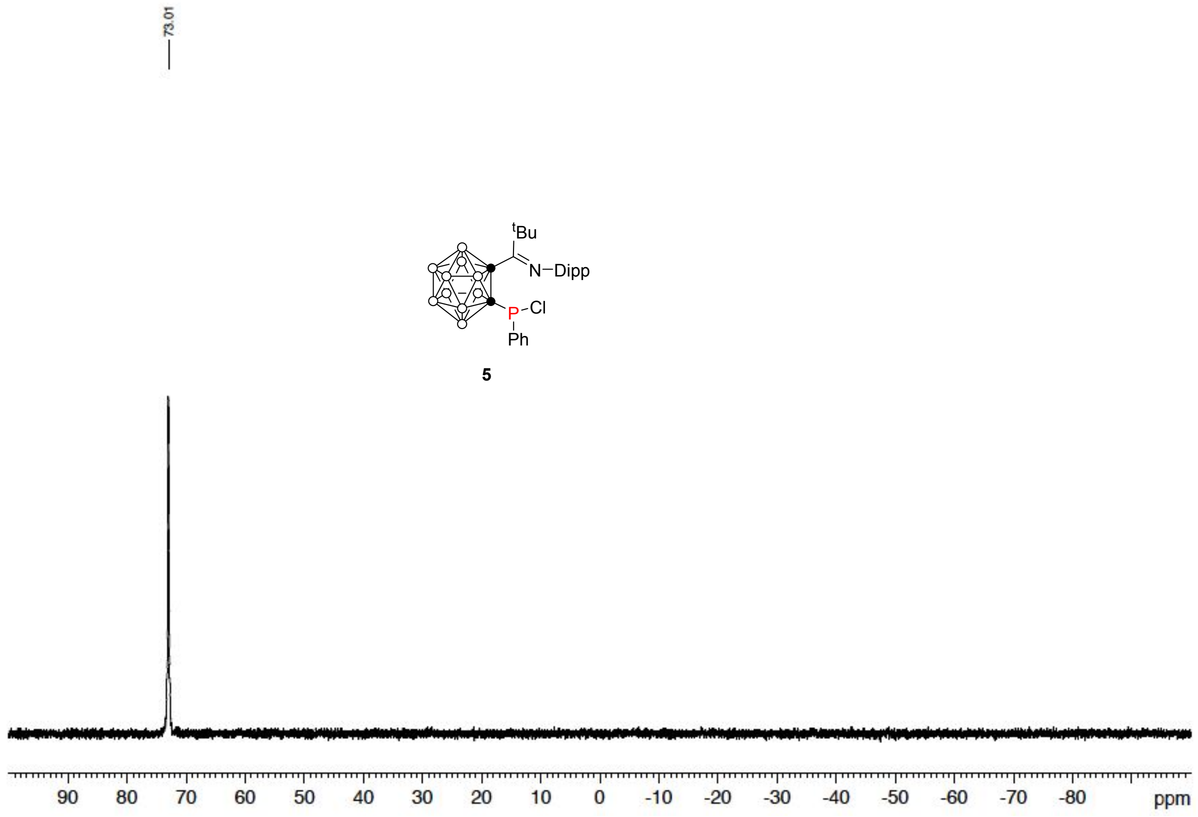

Figure S17. $^{31}P\{^{1}H\}$ NMR spectrum of **5** in CD_2Cl_2.

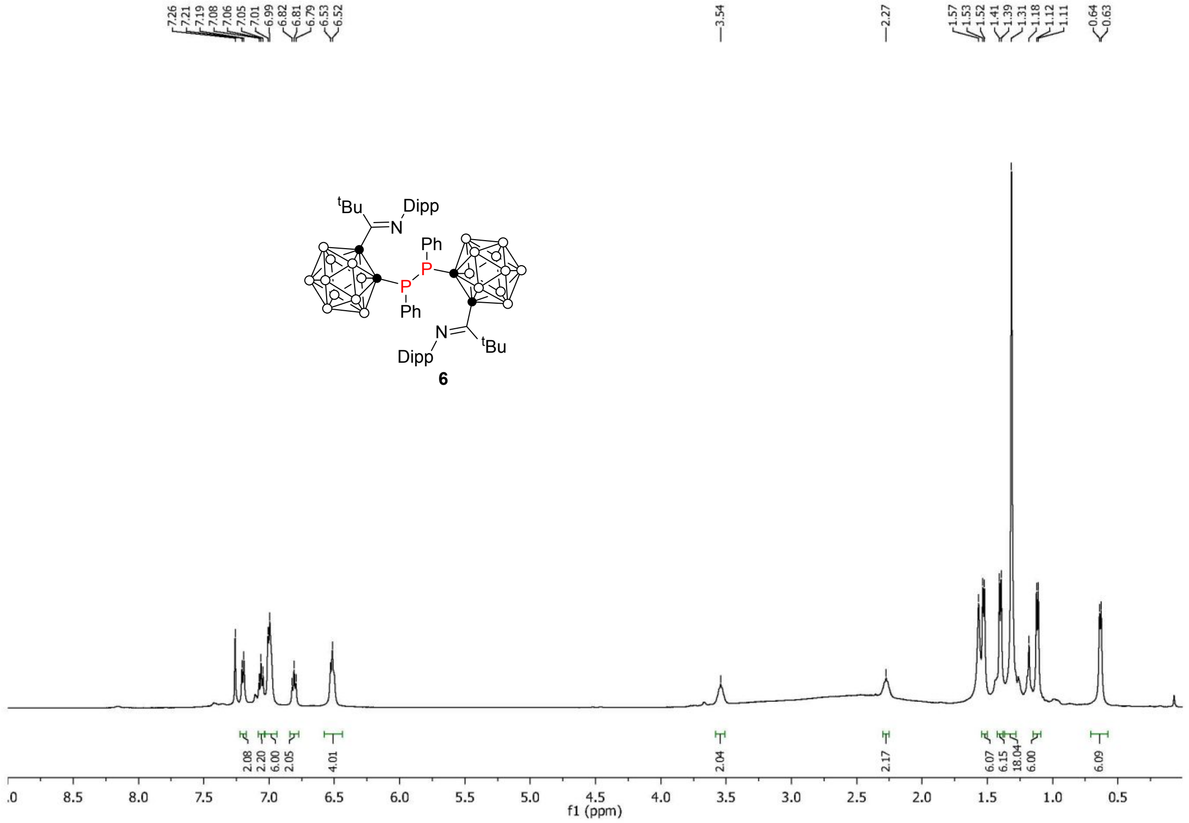

Figure S18. ^{1}H NMR spectrum of **6** in $CDCl_3$.

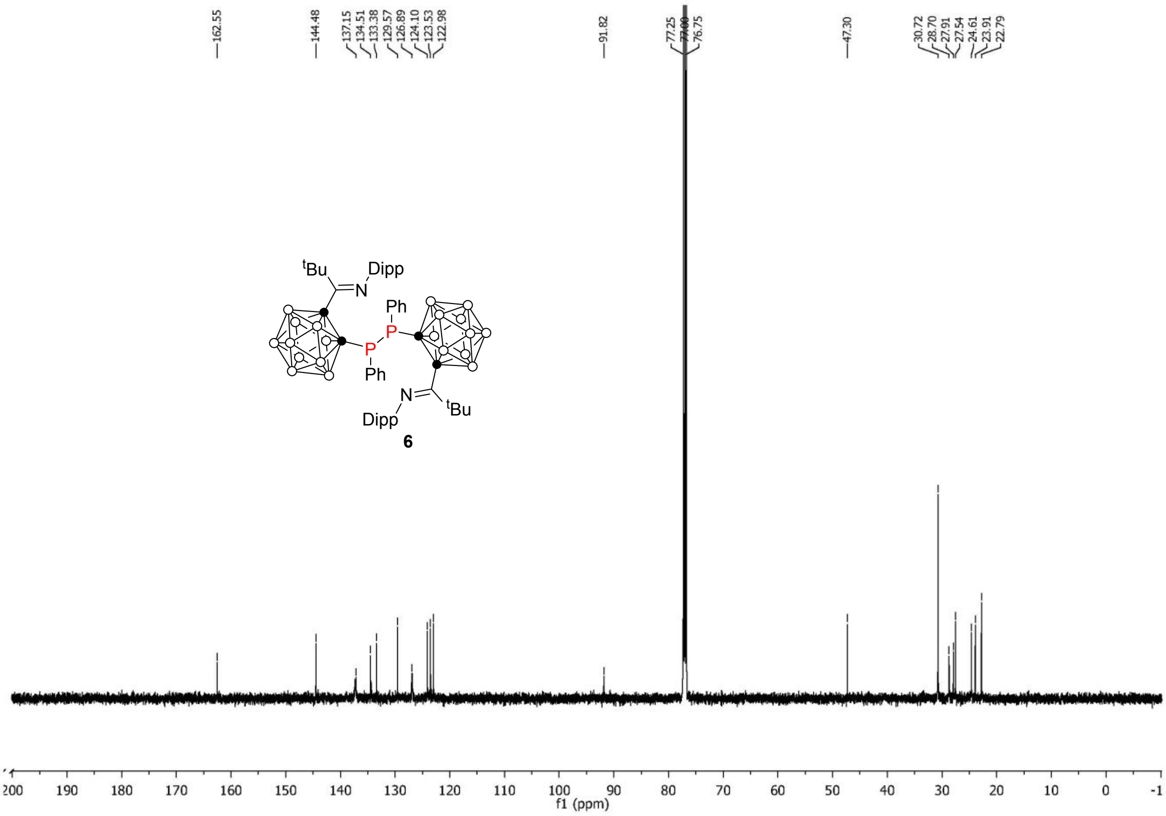

Figure S19. $^{13}C\{^{1}H\}$ NMR spectrum of **6** in $CDCl_3$.

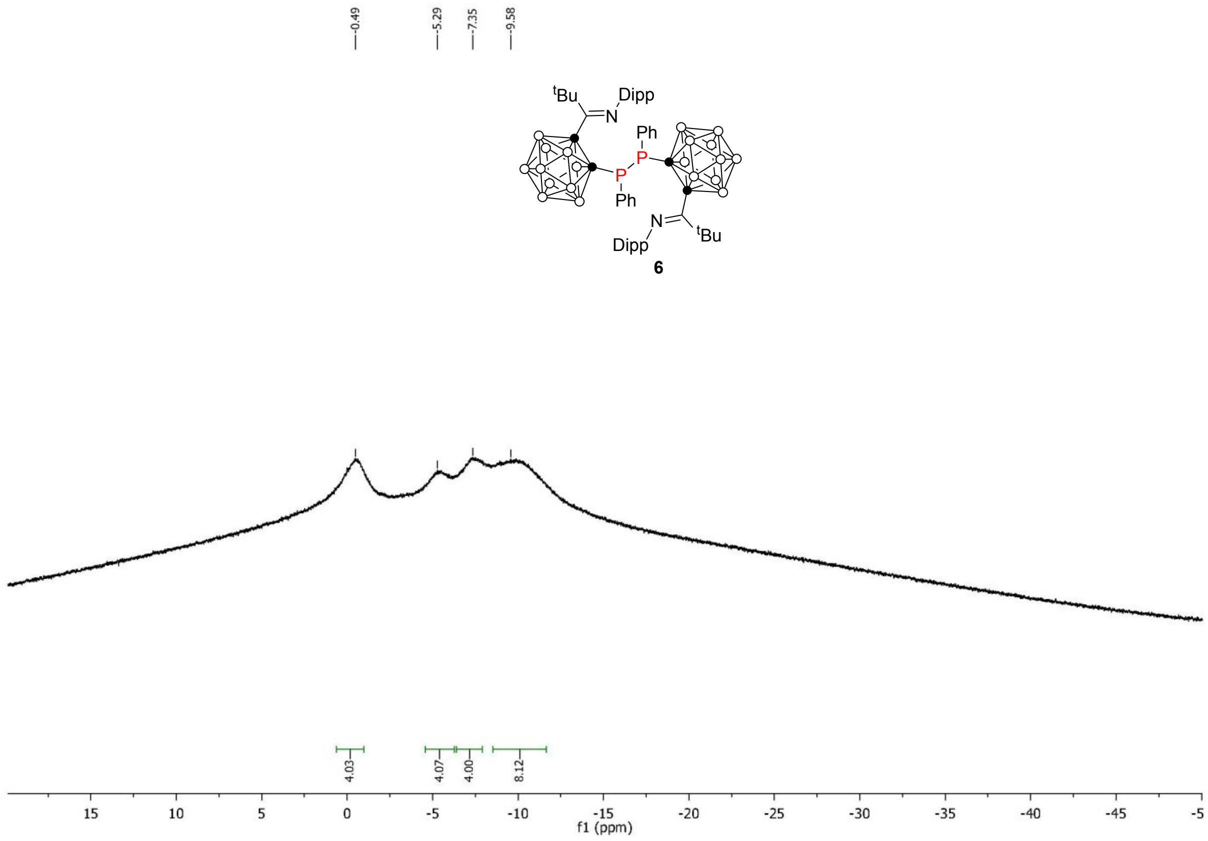

Figure S20. $^{11}B\{^{1}H\}$ NMR spectrum of **6** in $CDCl_3$.

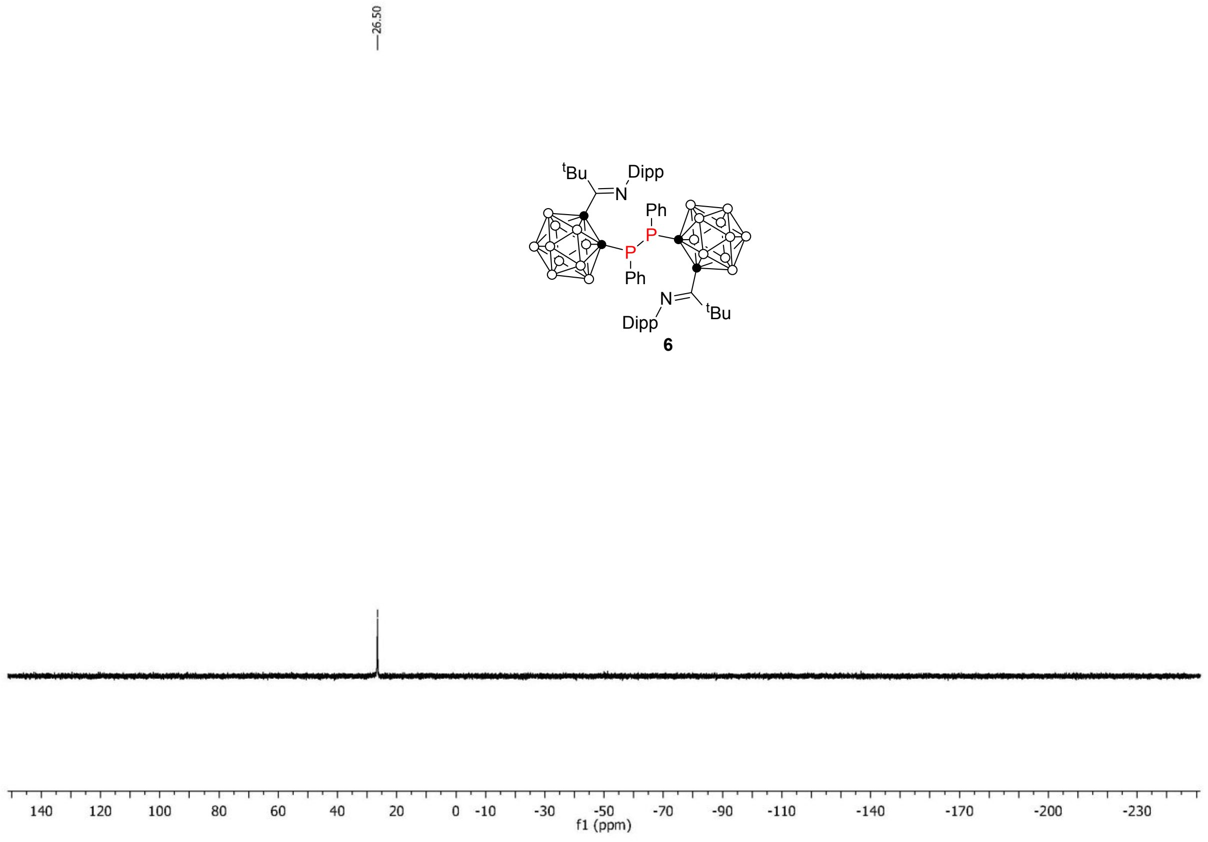

Figure S21. $^{31}P\{^{1}H\}$ NMR spectrum of **6** in $CDCl_3$.
